# Alpha-Lipoic Acid Prevents Side Effects of Therapeutic Nanosilver without Compromising Cytotoxicity in Experimental Pancreatic Cancer

**DOI:** 10.3390/cancers13194770

**Published:** 2021-09-24

**Authors:** Xuefeng An, Li Liu, Michael Schaefer, Bin Yan, Christian Scholz, Stefan Hillmer, Kangtao Wang, Yiqiao Luo, Huihui Ji, Jury Gladkich, Ingrid Herr

**Affiliations:** 1 Department of General, Visceral & Transplant Surgery, Molecular OncoSurgery, Section Surgical Research, University of Heidelberg, 69120 Heidelberg, Germany; Xuefeng.An@stud.uni-heidelberg.de (X.A.); l.liu@uni-heidelberg.de (L.L.); michael.schaefer@exchi.uni-heidelberg.de (M.S.); Bin.Yan@stud.uni-heidelberg.de (B.Y.); kangtao.wang@stud.uni-heidelberg.de (K.W.); luoyiqiaosc@163.com (Y.L.); huihui.ji@uni-heidelberg.de (H.J.); gladkich@uni-heidelberg.de (J.G.); 2 Institute of Earth Sciences, University of Heidelberg, 69120 Heidelberg, Germany; christian.scholz@geow.uni-heidelberg.de; 3 Electron Microscopy Core Facility, University of Heidelberg, 69120 Heidelberg, Germany; stefan.hillmer@urz.uni-heidelberg.de

**Keywords:** pancreatic cancer, silver nanoparticle, α-lipoic acid, chelation, BCAT1, oxidative stress, cytotoxicity

## Abstract

**Simple Summary:**

Pancreatic ductal adenocarcinoma is among the most aggressive malignancies and improved treatment options are urgently needed. Silver nanoparticles are suggested as potent antitumor agents, but the side effects of silver overdoses may limit the application. The natural anti-oxidant α-lipoic acid might prevent these side effects. We synthesized nanosilver and used it to treat several pancreatic cancer cells and normal cells in the presence or absence of α-lipoic acid. Silver selectively eliminated pancreatic cancer cells and α-lipoic acid supported the cytotoxicity, whereas benign cells largely resisted. α-Lipoic acid formed complexes with silver particles and reduced silver-induced formation of reactive oxygen species, mitochondrial damage and liver toxicity. Our data suggest that nanosilver application in the presence of α-lipoic acid is safe and effective in the treatment of pancreatic cancer.

**Abstract:**

Silver nanoparticles (AgNPs) have attracted attention in cancer therapy and might support the treatment of pancreatic ductal adenocarcinoma (PDAC). Silver is in clinical use in wound dressings, catheters, stents and implants. However, the side effects of systemic AgNP treatment due to silver accumulation limit its therapeutic application. We evaluated whether the antioxidant and natural agent α-lipoic acid might prevent these side effects. We synthesized AgNPs using an Ionic-Pulser^®^ Pro silver generator and determined the concentration by inductively coupled plasma–optical emission spectrometry. The effect of α-lipoic acid was examined in four PDAC and two nonmalignant cell lines by MTT, FACS analysis, TEM, xenotransplantation and immunohistochemistry. The viability of PDAC cells was nearly totally abolished by AgNP treatment, whereas nonmalignant cells largely resisted. α-Lipoic acid prevented AgNP-induced cytotoxicity in nonmalignant cells but not in PDAC cells, which might be due to the higher sensitivity of malignant cells to silver-induced cytotoxicity. α-Lipoic acid protected mitochondria from AgNP-induced damage and led to precipitation of AgNPs. AgNPs reduced the growth of tumor xenografts, and cotreatment with α-lipoic acid protected chick embryos from AgNP-induced liver damage. Together, α-lipoic acid strongly reduced AgNP-induced side effects without weakening the therapeutic efficacy.

## 1. Introduction

Pancreatic ductal adenocarcinoma (PDAC) is one of the most aggressive common malignancies and is characterized by late diagnosis, early metastasis, and high therapy resistance. Despite worldwide efforts, therapeutic options are limited. PDAC remains the fourth leading cause of cancer-related deaths with an estimated 47,050 deaths in the US in 2020, which account for 8.0% of all cancer deaths, and 57,600 new cases, which account for 3.0% of all new cancer cases [1]. Therefore, improved therapeutic options are urgently required.

Numerous recent studies describe silver nanoparticles (AgNPs) as potent antitumor agents [2,3], e.g., for experimental treatment of PDAC [4]. Silver is known as one of the most effective killers of any type of fungus, bacterium and virus [5], including the SARS-CoV-2 virus (COVID-19) [6], and has been used since ancient times for its antimicrobial properties. For example, silver coins preserved milk, silver vessels kept drinking water fresh, and silver plates on wounds prevented infection [5,7]. Hippocrates (400 BCE) used silver powder for the treatment of ulcers and infectious diseases, and Paracelsus used silver nitrate salt for the treatment of wounds [5,7]. During the Middle Ages, upper social class families used everyday household plates and cutlery made from silver, which might have protected them from the bubonic plague [8]. The price was permanent bluish-gray skin after significant exposure to silver, a medical condition called argyria. Silver particles with diameters of 7 to 9 nm have been used in medications since 1889 and are known as “colloidal silver” [9]. The treatment of patients with antimicrobial silver was largely discontinued in the 1940s after the discovery of penicillin, for which Sir Alexander Fleming, a Scottish researcher, was credited in 1928. However, Fleming already recognized the potential of bacteria to develop resistance. Nanosilver is discussed to circumvent the increasing resistance of bacteria to antibiotics because it is effective even against methicillin-resistant *Staphylococcus aureus* (MRSA) [10,11]. In modern medical applications, silver is routinely used in dressings to treat burns, skin wounds and skin infections; silver nitrate is used for Crede prophylaxis to prevent chlamydia or gonococcal conjunctivitis in newborns [12], and aortic, heart, biliary or ureteral stents are coated with silver nanoparticles to prevent infection and stent blockade by biofilms [13,14]. For inoperable, obstructing PDAC, palliative, silver-coated biliary stents are applied [13].

In addition to pure metallic silver (Ag), there are silver salts, silver nitrates, silver sulfates, silver zeolite and silver protein complexes up to silver nanoparticles (AgNPs). The common working principle of any form of silver seems to be the release of monoatomic silver ions (Ag^+^) [5]. A suspension of silver-containing particles with sizes ranging from 1 to 1000 nm is loosely defined as colloidal silver [15]. Silver nanoparticle suspensions are most often mixtures of silver ions, nanoparticles and aggregated nanoparticles [16]. The nanosilver particle sizes range from 1 to 100 nm [17]; they have a greater surface area and produce more silver ions than complex silver reagents. It is thought that the antitumor and antibacterial activities of silver nanoparticles depend on the silver ions, which are released from the nanoparticle surface [18].

The molecular mechanisms involved in the cytotoxicity of AgNPs against cancer cells are still under investigation. A direct interaction of AgNPs with cellular proteins, DNA, antioxidants, enzymes, membranes and organelles is suggested to converge into cell death [5,19]. Alternatively, AgNPs are thought to indirectly influence cellular functions by the generation of reactive oxygen species (ROS) and oxidative stress, which then causes DNA damage, protein modification and degradation, metabolic toxicity and cellular dysfunction, which finally lead to cell death [5,20]. To date, the only available information on silver effects on pancreatic cancer cells is that exposure of the established PDAC cell line PANC-1 to AgNPs led to an altered mitochondrial ultrastructure and increased ROS generation [4].

Side effects, such as agyria, have been observed after long-term ingestion of high amounts of colloidal silver [21,22]. A case report described agyria after five years of occasional ingestion of colloidal silver, and the estimated daily mean amount was 700 mL of a 0.15 µg/mL (=0.15 ppm) colloidal silver solution, which corresponds to a total silver amount of 0.2 g silver ingested in 5 years [21]. It is assumed that agyria might be due to the stimulation of melanocytes by deposited silver or to a sunlight-induced reduction of the initially colorless silver in the dermis [23,24]. Additionally, silver deposition following oral administration has been detected in the liver, kidneys, brain, spleen, blood and small intestine [25]. Besides, neurotoxicity, changes in liver enzymes, weight loss, hypoactivity and immunological effects are described following excessive administration of silver nanoparticles [17,26].

The naturally occurring antioxidant and anti-inflammatory agent α-lipoic acid is present in organ meats, spinach, broccoli, tomato, peas, Brussels sprouts and rice [27]. α-Lipoic acid is also synthesized by the human body within the mitochondria, which explains its important function as a cofactor of pyruvate and alpha-ketoglutarate dehydrogenase in energy metabolism. In addition, α-lipoic acid was demonstrated to reverse the toxicity of mercury derived from dental amalgams [28], excess copper in Wilson disease [29], heavy metal intoxication, toxic mushroom poisoning, diabetic polyneuropathy and liver cirrhosis [28]. Due to its lipophilic properties, α-lipoic acid penetrates cell membranes and reaches high intracellular concentrations within 30 s of administration [30]. α-Lipoic acid forms complexes with manganese (Mn^2+^), zinc (Zn^2+^), cadmium (Cd^2+^), lead (Pb^2+^), cobalt (Co^2+^), nickel (Ni^2+^) and iron (Fe^2+^) [31]. A recent publication hints at the possibility that α-lipoic acid also forms complexes with silver ions (Ag^+^) because it capped AgNPs on human gingival fibroblasts while maintaining its antimicrobial effect for oral applications [32].

In the present study, we examined the effect of α-lipoic acid on the cytotoxicity of AgNPs on human PDAC cell lines and benign cells. We found that AgNPs quickly killed PDAC cell lines, whereas benign cells largely resisted. Our results demonstrate that α-lipoic acid forms complexes with silver nanoparticles and ions and reduces AgNP-induced ROS formation, mitochondrial damage and liver toxicity. At the same time, the high therapeutic cytotoxicity of nanosilver to PDAC cells was maintained.

## 2. Materials and Methods

### 2.1. Cell Lines

The human PDAC cell lines BxPc-3, PANC-1 and MIA-PaCa2 and the nonmalignant human pancreatic ductal cell line CRL-4023 (hTERT-HPNE-immortalized) were purchased from the American Type Culture Collection (ATCC, Manassas, VA, USA). Immortalized human hepatic stellate LX-2 cells were purchased from Merck (Darmstadt, Germany). Gemcitabine-resistant BxGEM cells were selected by continuous gemcitabine treatment of parental BxPc-3 cells as described [33]. BxPc-3, BxGEM, PANC-1, MIA-PaCa2 and LX-2 cells were cultured in Dulbecco’s modified Eagle’s medium with high glucose supplemented with 100 µg/mL fetal bovine serum (both from Sigma-Aldrich, Taufkirchen, Germany) and 1 mM HEPES (PAA Laboratories, Posching, Austria). CRL-4023 cells were cultured in Dulbecco’s modified Eagle’s medium without glucose (Thermo Fisher, Frankfurt, Germany) and Medium M3 Base (Incell Corp, San Antonio, TX, USA) at a ratio of 3:1 with 2 mM L-glutamine, adjusted to 1.5 g/L sodium bicarbonate, and supplemented with 5% fetal bovine serum, 10 ng/mL human recombinant EGF, 750 ng/mL puromycin (all from Sigma-Aldrich, Taufkirchen, Germany) and 5.5 mM D-glucose (Merck Darmstadt, Germany). All cell lines were cultured at 37 °C in a humidified atmosphere of 95% O_2_ and 5% CO_2_, and they were authenticated by Multiplexion GmbH (Heidelberg, Germany) and by their typical morphology throughout the culture. To maintain authenticity of the cell lines, frozen stocks were prepared from initial stocks, and every three months, a new frozen stock was used for the experiments. Mycoplasma-negative cultures were ensured by monthly testing by PlasmoTest™ (InvivoGen, San Diego, CA, USA).

### 2.2. Reagents

α-Lipoic acid (≥99%) (Sigma-Aldrich, Taufkirchen, Germany) was dissolved in ethanol to a 200 mM stock solution and stored in aliquots at −20 °C. Each aliquot was used only once immediately after thawing. The final concentrations of the solvents in media were 0.5% or less.

### 2.3. AgNP Synthesis

AgNPs were produced by the use of the IDEAL-Pulser Basic S silver generator (Pestalozzi-Apotheke, Lörrach, Germany). In short, 200 mL H_2_O_bidest_ was boiled and used to fill a heat-resistant laboratory glass beaker; then, the generator was placed on top of the beaker so that the 8 cm-long and 5 mm-thick electrodes of pure silver > 99.99% and a distance of 1 mm between each electrode were almost completely immersed in water. During the synthesis, the electrode sludge was wiped off with a lint-free cellulose cloth every 15 min. Finally, the AgNP solution was filtered through a 0.2 µm syringe filter (GE Healthcare, Frankfurt, Germany) and transferred to an amber glass bottle for short-term storage.

### 2.4. Determination of Silver Ion Concentration

The conductivity of AgNP solutions was measured by the use of an HP8753C network analyzer in combination with an HP86046 A S-parameter test set (Hewlett-Packard, Palo Alto, CA, USA) for microwave reflectometry. The conductivity was determined by measuring the complex reflection coefficient on an open coaxial line immersed in the AgNP solution as described [34]. Briefly, at 10 MHz, the conductivity of an aqueous solution was calculated using the imaginary part of the complex dielectric permittivity, which was obtained from the measured reflection coefficient. Assuming that only Ag^+^ and OH^−^ ions are produced by pulsing H_2_O_bidest_, the conductivity was considered to be the difference in the conductivity of the silver solution minus that of pure water. The calculation of the silver concentration was as follows:

(1) $$\sigma_{Ag}^{\prime }\left(10\;\mathrm{MHz}\right)-\sigma_{H2o}^{\prime }\left(10\;\mathrm{MHz}\right)=e\left(Z_{Ag}\mu_{Ag}n_{Ag}+Z_{OH}\mu_{OH}n_{OH}\right)$$

with the elementary charge $e=1.6\times 10^{-19}C$, charge number $Z_{Ag}$ = $Z_{OH}$ = 1, ion mobility $\mu_{Ag}=6.42\times 10^{-8}\frac{m^{2}}{Vs}$, $\mu_{OH}=2.052\times 10^{-7}\frac{m^{2}}{Vs}$ at 18 °C, and number density $n_{Ag}$ = $n_{OH}$ of the Ag^+^ and OH^−^ ions.

The conductivity measurements were performed at 25 °C, thus the ion mobilities had to be calculated for this temperature using Equation (2).

(2) $$\mu_{i}\left(25\;{}^{\circ}C\right)=\mu_{i}\left(18\;{}^{\circ}C\right)\left(1+\alpha_{i}\left(25\;{}^{\circ}C-18\;{}^{\circ}C\right)\right)$$

and *i* = Ag, OH, $\alpha_{Ag}=0.0209\frac{1}{K}$, $\alpha_{OH}=0.0206\frac{1}{K}$.

From Equation (1) we calculated the number density $n_{Ag}$, which was given in ppm, based on the silver molar weight of 107.87 g/mol.

### 2.5. Quantification of Total Silver Concentration

To determine the intracellular silver concentration, the cells were treated with 1.4 ppm AgNPs for 24 h, and then the cells were detached from the cell culture plate by the use of trypsin and washed with PBS. To detect the silver concentration in liver tissues, the livers were resected from 10 embryos of each group, and the tissues were minced with sterile scissors. Then, the cells and livers were dissolved and acidified in 400 µL of 65% HNO_3_ for 24 h at 65 °C. H_2_O_bidest_ was added to a total volume of 4 mL to each sample, and the silver concentration was determined by plasma optical emission spectrometry (ICP-OES) at a wavelength of 328.068 nm using an Agilent 720 ICP-OES device (Agilent, Santa Clara, CA, USA). To assess the amount of silver deposited in each cell in vitro or per gram of liver tissue in vivo, the obtained results of each group were divided by the cell number or the weight of lysed liver (in grams). A conical nebulizer with a cyclone chamber served as the sample introduction system (Agilent). All samples were prepared and analyzed in duplicate.

### 2.6. Transmission Electron Microscopy (TEM)

Round, sterile coverslips (Carl Roth, Karlsruhe, Germany) were placed in Greiner Cell Star^®^ 24-well plates (Merck, Darmstadt, Germany), and 5 × 10^4^ cells were seeded. Twenty-four hours later, the cells were treated, with the final concentrations in the medium containing 1.4 ppm AgNPs w and w/o 1 mM α-lipoic acid or were left untreated in the control. Twenty-four hours later, the cells were fixed for 30 min at room temperature with 2.5% glutaraldehyde, 2% sucrose in 50 mM sodium-cacodylate buffer (pH 7.2; Sigma-Aldrich, Taufkirchen, Germany) supplemented with 50 mM KCl, 2.6 mM MgCl_2_ and 2.6 mM CaCl_2_. Afterwards the samples were washed 5 × 2 min with 50 mM cacodylate buffer and post fixed for 40 min (at 4 °C in the dark) with 2% osmium tetroxide in 50 mM sodium-cacodylate buffer. After 3 washing steps with H_2_O_bidest_ (5 min each), the coverslips were en bloc stained with 0.5% aqueous uranyl acetate for 30 min at room temperature in the dark. Samples were dehydrated in a graded ethanol series (from 40% to 100%) at RT and finally coverslips were placed on capsules filled with Spurr-resin and polymerized for 24 to 48 h at 60 °C. Embedded samples were sectioned using a Reichert Ultracut S ultramicrotome (Leica Instruments, Vienna, Austria) to a thickness of 70 nm. Post-staining was done with 3% uranyl acetate in H_2_O_bidest_ and lead citrate. Imaging was done at a Jeol JEM-1400 (Jeol Ltd., Tokyo, Japan), operating at 80 kV, equipped with a 4 k × 4 k digital camera (TEMCAM F416, TVIPS, Gauting, Germany) using EMMenue4 for taking micrographs. Characterization of silver nanoparticles was done by dropping the suspension onto a formvar coated grid using a micropipette. After a few seconds the grid was briefly washed with a drop of H_2_O_bidest_ and air dried afterwards. Samples were imaged without further staining.

### 2.7. Measurement of E. coli Growth

The *Escherichia coli* (*E. coli*) bacterial strain OP50 was cultured in LB medium overnight and was then diluted to an OD_600_ of ~0.1. Then, 0.1 mL of the bacterial solution was pipetted into single wells of a 96-well plate containing LB medium plus AgNPs at final concentrations of 1.4 ppm, 2.8 ppm, 4.2 ppm, 5.6 ppm or 7.0 ppm or the solvent H_2_O_bidest_ alone, followed by culture at 37 °C for 24 h. Then, the OD_600_ was measured in 60-min intervals through the use of a Helios Delta spectrophotometer (Thermo Spectronic, London, UK) for a period of 8 h. The growth curve was created using GraphPad Prism 6.0 software (GraphPad Software Inc., San Diego, CA, USA).

### 2.8. Measurement of Cell Viability by MTT Assay

A total of 5 × 10^3^ cells/100 µL cell culture medium per well of a 96-well plate was seeded (*n* = 8 per group). Twenty-four hours later, the cells were treated with 1.4 ppm AgNPs w and w/o 0.5 mM or 1 mM α-lipoic acid were left untreated in the control. Following incubation for 24 h, 10 µL 3-(4,5-dimethylthiazol-2-yl)-2,5-diphenyltetrazolium bromide (MTT) (Sigma-Aldrich, Taufkirchen, Germany) was added to each well, followed by incubation at 37 °C for 4 h until the formation of violet formazone crystals became visible. Then, 200 µL of DMSO was added to each well and incubated with gentle shaking at 37 °C for 15 min. The absorbance was measured at 560 nm using a Biotek EL800 microplate reader (BioTek Instruments, Winooski, VT, USA) with a reference wavelength of 630 nm. The cell viability was calculated based on the optical density. For data analysis, the value of the DMSO control background was subtracted. The calculated value of the control of each cell line was set to 100%.

### 2.9. Determination of Intracellular ROS by H2DCFDA-Cellular ROS Assay

The cells were seeded into 6-well plates at a density of 1.2 × 10^5^ per well. Twenty-four hours later, the cells were treated with 1.4 ppm AgNPs w and w/o 1 mM α-lipoic acid or were left untreated in the control for 24 h. After discarding the culture medium, the cells were washed once with 5 mL PBS, and then the cells were detached by the use of trypsin-EDTA (Thermo Fisher, Frankfurt, Germany), followed by washing in PBS. The cell pellet was diluted in 300 µL of the buffer provided in the H2DCFDA-Cellular ROS Assay Kit (Abcam, Berlin, Germany) and incubated in 10 µM cell-permeant 2′,7′-dichlorodihydrofluorescein diacetate for 0.5 h at 37 °C. The fluorescence of DCF, which reflects the oxidized form of H2DCFDA, was measured with a FACSCanto II flow cytometer (BD Biosciences, Heidelberg, Germany), and the excitation and emission wavelengths of 480 and 525 nm were evaluated from 10,000 individual cells per measured value.

### 2.10. Staining of Mitochondria

The cells were seeded into 24-well plates at a density of 5 × 10^4^ per well. Twenty-four hours later, the cells were treated with 1.4 ppm AgNPs w and w/o 1 mM α-lipoic acid or were left untreated in the control. Twenty-four hours later, the cell culture medium was removed, and the cells were stained according to the instructions of the Mitochondrial Staining Kit—Red Fluorescence—Cytopainter (Abcam, Berlin, Germany). Randomly chosen fields were examined at 400× magnification using a Leica DMRB fluorescence microscope. Images were captured using a SPOTTM FLEX 15.2 64 Mp shifting pixel digital color camera (Diagnostic Instruments, Inc., Sterling Heights, MI, USA) and analyzed with SPOT Advanced 4.6 software (SPOT Imaging^TM^, Sterling Heights, MI, USA).

### 2.11. Detection of Apoptosis and Necrosis by Flow Cytometry

The cells were seeded into 6-well plates at a density of 5 × 10^4^/mL per well. Twenty-four hours later, the cells were treated with 1.4 ppm AgNPs w and w/o 1 mM α-lipoic acid or were left untreated in the control. Twenty-four hours later, the cells were detached by the use of trypsin without EDTA (Thermo Fisher, Frankfurt, Germany), followed by washing in PBS. Then, the cells were resuspended in 1× Annexin V Binding Buffer (BD Bioscience, Heidelberg, Germany), and 1 × 10^6^ cells were collected and incubated with 1 µM Annexin V-FITC (BD Bioscience, Heidelberg, Germany) for 0.5 h at room temperature. After washing three times with 1× Annexin V Binding Buffer, the cells were incubated in 200 µL 1× Annexin V Binding Buffer supplemented with 2 µL 7-aminoactinomycin (7-AAD; BD Bioscience, Heidelberg, Germany) for 20 min at room temperature. Subsequently, the cells were washed and resuspended in 300 µL 1× Annexin V Binding Buffer and stored in the dark on ice for up to 0.5–1 h until FACS measurement. Annexin V-FITC (excitation/emission: 494/518 nm) binds to phosphatidylserine exposed by apoptotic cells on the cell surface. 7-AAD is a fluorescent intercalator that experiences a spectral shift after DNA binding, and 7-AAD/DNA complexes can be excited by a laser at 488 nm with a maximum emission wavelength of 647 nm. In general, 7-AAD is excluded from living cells but binds to the DNA of late apoptotic or necrotic cells. The fluorescence of cells stained with annexin V-FITC/7-AAD was measured with a FACS Canto II flow cytometer (BD Biosciences, Heidelberg, Germany). The excitation and emission wavelengths of 488 and 650 nm were evaluated from 10,000 individual cells per measured value.

### 2.12. Evaluation of Growth and Liver Morphology of Chick Embryos

Fertilized eggs from genetically identical hybrid Lohman brown chickens were obtained from a local ecological hatchery (Geflügelzucht Hockenberger, Eppingen, Germany). The eggs were washed with 70% ethanol and placed in sterile 37.8 °C incubators in a vertical position with the pointed side of the eggs downwards. On day 4 of chick development, a 1-cm^2^ hole was cut with scissors into the eggshell to check the viability of the embryo. Afterward, the eggs with live embryos were replaced in the incubator and kept in a horizontal position. Eggs with dead embryos were excluded from the experiment. From developmental day 12 to day 17, the chick embryos were treated by dropping 200 µL or 400 µL of a 14 ppm AgNP solution, 200 µL or 400 µL of a 0.5 mM α-lipoic acid solution, or both solutions together on the chorioallantoic membrane (CAM). Control eggs received 400 µL H_2_O_bidest_ only. On developmental day 18, the chick embryos were sacrificed by injection of 10 µL of a 25 mg/mL ketanest^@^ solution (Pfizer Pharma PFE GmbH, Berlin, Germany) into a CAM vessel. Each embryo was photographed and weighed, and the livers were resected and incubated in 10% formalin. Then, the liver tissue was embedded in paraffin, and 3–5 µm tissue sections were prepared by the use of a HistoCore Rotary Microtome (LEICA RM2155, Leica Biosystems, Wetzlar, Germany), placed on Superfrost slides (Menzel-Gläser, Braunschweig, Germany), and dried overnight. The sections were deparaffinized by consecutive immersion in petroleum ether, 99% ethanol, 95% ethanol and 70% ethanol, followed by 3× washing with PBS. The liver sections prepared in this way were subsequently stained with hematoxylin (Carl Roth, Karlsruhe, Germany) for 1 min, washed 3× with H_2_O_bidest_ and counterstained with eosin-phloxine (Sigma-Aldrich Chemie GmbH, Schnelldorf, Germany) for 30 s. Then, the sections were dehydrated by 2× washing with 95% EtOH for 5 min and mounted in a xylene-based mounting medium (SouthernBiotech, Birmingham, AL, USA). The stained liver tissues were evaluated under a Leica DMRB fluorescence microscope using SPOT Advanced Version 4.6 software (SPOT Imaging^TM^, Sterling Heights, MI, USA).

### 2.13. Tumor Xenotransplantation of The CAM of Fertilized Chicken Eggs

Fertilized chicken eggs were obtained and incubated as described above. On day 8 of chick development, 1 × 10^6^ MIA-PaCa_2_ cells were mixed with Corning™ Matrigel™ Matrix (Corning Life Science BV, Amsterdam, The Netherlands) at a ratio of 1:1 to a total volume of 50 µL. Coverslips (Carl Roth, Karlsruhe, Germany) were punched, and the resulting rings were placed on the CAM. After gently scratching the surface of the CAM in the center of the coverslip with a 0.4 mm × 19 mm stainless-steel needle (BD Microlance™, Louth, Ireland), the mixture of cells and Matrigel™ was transplanted onto the wounded region. From developmental day 12 to day 17, 400 µL of a 14 ppm AgNP solution, 400 µL of a 0.5 mM α-lipoic acid solution or both solutions together were dropped onto CAM vessels that supplied the MIA-PaCa2 xenograft tumors. Control eggs received 400 µL H_2_O_bidest_ only. At day 18, the embryos were gently and humanely euthanized by injection of 10 µL of a 25 mg/mL ketanest^@^ solution (Pfizer Pharma PFE GmbH, Berlin, Germany) into a CAM vessel, followed by tumor resection and the evaluation of the tumor volumes 3-dimensionally by a USB microscope camera (eScope, Oitez, Hong Kong) and digital image editing using a customized mount.

### 2.14. mRNA Microarray Profiling

LX-2 cells were seeded into 6-well plates at a density of 5 × 10^4^ mL per well. Twenty-four hours later, the cells were treated with 1.4 ppm AgNPs w and w/o 1 mM α-lipoic acid at final concentrations or were left untreated in the control, followed by incubation for an additional 24 h. Then, the cells were harvested, and mRNA was isolated by using an RNeasy Mini Kit (QIAGEN, Hilden, Germany) according to the manufacturer’s instructions. Microarray analysis was performed at the Microarray-Analytic Center of the Medical Faculty Mannheim by using Clariom™ D Assays (Thermo Fisher Scientific, Darmstadt, Germany).

### 2.15. Gene Set Enrichment Analysis (GSEA)

To determine the interplay between α-lipoic acid and AgNPs and their relationship to pathway regulation, GSEA was performed (software version 4.1.0). A list of preranked genes, which was generated from our gene microarray based on a score calculated as the -log10 of the *p* value multiplied by the sign of the fold change, was tested in predefined annotated gene sets (C1–C7 collections) from the Molecular Signatures Database (MSigDB v7.4) to find significant differences between two phenotypes. A heatmap was created by using the free software environment for statistical computing and graphics R Studio (https://rstudio.com/products/rstudio/) (accessed on 1 March 2021).

### 2.16. Western Blot Analysis

A total of 1 × 10^5^ cells per well of a 6-well plate were seeded. Twenty-four hours later, the cells were cultured in a medium with 1.4 ppm AgNPs in the presence or absence of α-lipoic acid or were left untreated in the control. Following incubation for 24 h, the cells were washed, and proteins were harvested by the use of a RIPA lysis buffer (both from Abcam, Cambridge, UK). Western blot analysis was performed using a semidry blotting system according to a standard protocol. Primary antibodies were rabbit polyclonal antibody against BCAT1 (Abcam, Cambridge, UK, ab197941) and rabbit monoclonal antibody against GAPDH (Cell Signaling Technology, Danvers, MA, USA, Cat# 2118, RRID: AB_561053). IRDye^®^ infrared dye-conjugated secondary antibodies (LI-COR Biosciences, Bad Homburg, Germany) were used. The infrared intensity was measured with an Odyssey CLx Infrared Imaging System (LI-COR). 

### 2.17. Statistical Analysis

The quantitative data are presented as the means ± SD from at least three independent experiments, which were performed in triplicate or multiples thereof. Statistically relevant group sizes of at least 10 eggs per group were chosen for in vivo experiments. Differences between two groups were assessed by Student’s *t*-test and one-way ANOVA. SPSS 22.0 and GraphPad Prism 6 (San Diego, CA, USA) were used for statistical analysis, and a *p* < 0.05 was considered statistically significant.

## 3. Results

### 3.1. Synthesis and Characterization of Nanosilver

**Figure 1:**
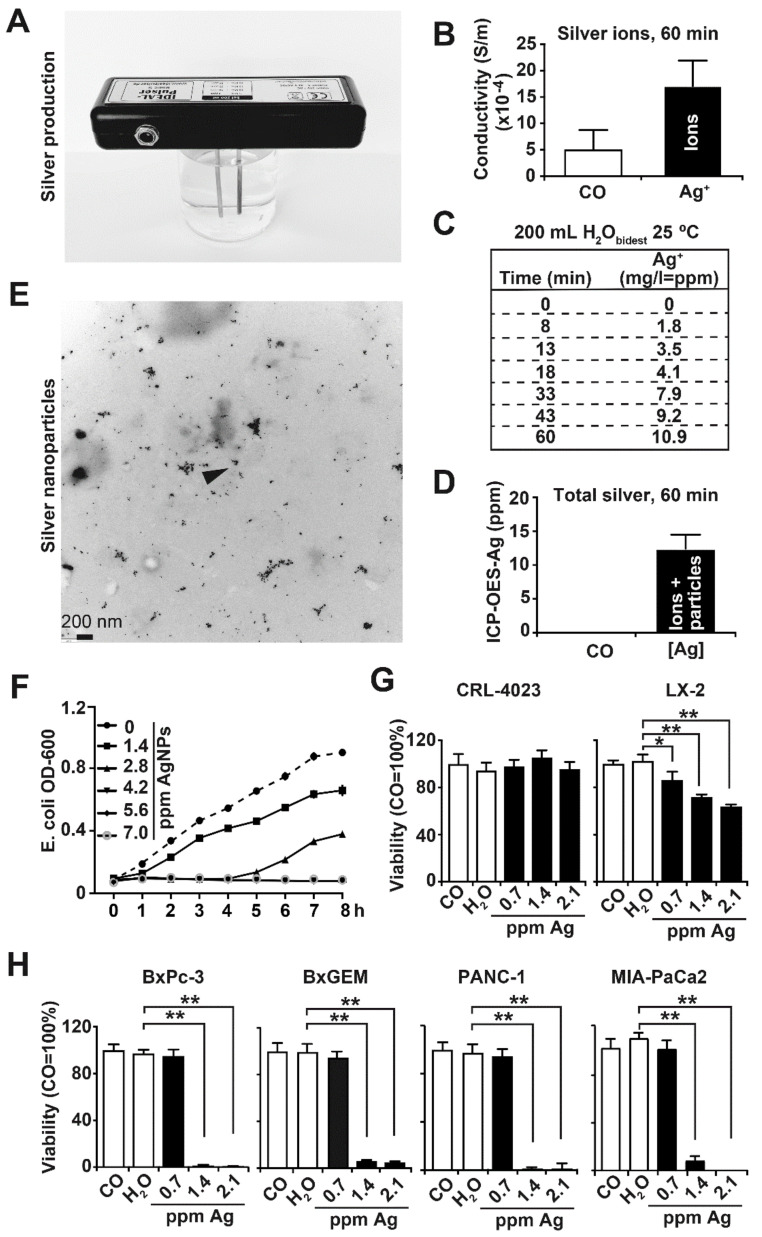
Generation of AgNPs and characterization. (**A**) Nanosilver particles were produced by pulsing 200 mL boiled H_2_O_bidest_ at 25 °C with the IDEAL-Pulser Basic S silver generator, and a representative picture is shown. (**B**) The conductivity of H_2_O_bidest_ (CO) and a AgNP (Ag) solution pulsed for 60 min was analyzed by a network analyzer, and the presence of conductive silver anions (Ag^+^) is given as Siemens per meter (S/m). (**C**) The concentration of Ag^+^ ions before and after pulsing for 8, 13, 18, 33, 43 and 60 min was determined by conductivity measurement and calculation. The values are provided as mg/L, which corresponds to parts per million (ppm). (**D**) The concentration of total silver, consisting of silver particles and Ag^+^ ions, was determined by plasma optical emission spectrometry (ICP-OES) in at least three independent experiments 60 min after pulsing. H_2_O_bidest_ served as the control (CO). (**E**) A 14 ppm AgNP solution was examined by TEM. The black dots indicate AgNPs, and the arrow points to a silver particle with a typical size of 9 nm. The visible larger clumps mainly result from aggregated particles. The scale bar indicates 200 nm. (**F**) *E. coli* OP50 bacteria were pipetted into single wells of a 96-well plate containing AgNPs at final concentrations of 1.4 ppm, 2.8 ppm, 4.2 ppm, 5.6 ppm or 7.0 ppm or the solvent H_2_O_bidest_ alone and were cultivated in LB medium for 24 h. The OD_600_ was measured in 60-min intervals with a spectrophotometer for a period of 8 h. (**G**) The nonmalignant human primary pancreatic duct cell line CRL-4023 and the hepatic stellate cell line LX-2 at a concentration of 5 × 10^3^ cells/100 µL per well of a 96-well plate were seeded. Twenty-four hours later, the cells were incubated in 100 µL cell culture medium with 5 µL of 14 ppm AgNP stock solution, resulting in a final AgNP concentration of 0.7 ppm. Likewise, 10 µL and 15 µL of a 14 ppm AgNP stock solution were added, resulting in final concentrations of 1.4 ppm and 2.1 ppm AgNPs, respectively. The control cells were left untreated (CO) or incubated in 100 µL medium containing 15 µL H_2_O_bidest_ (H_2_O). After incubation for 24 h, viability was determined by MTT assay. The data shown are derived from at least three independent experiments; the controls (CO) of each cell line were set to 100%. (**H**) The PDAC cell lines BxPC-3, BxGEM, PANC-1 and MIA-PaCa2 were treated and analyzed by MTT assay, as described above. The data are presented as the mean values, and standard deviations are given. * *p* < 0.05, ** *p* < 0.01.

Nanosilver was prepared from 200 mL H_2_O_bidest_ by the use of at least 99.99% pure silver electrodes and the Ionic-Pulser^®^ Pro silver generator (Figure 1A). The presence of Ag^+^ ions before and after pulsing was determined by measuring the conductivity of the aqueous silver solution with a network analyzer. We found an intensity of 15 × 10^−4^ Siemens per meter 60 min after pulsing (Figure 1B). The concentration of Ag^+^ ions was calculated by subtracting the conductivity of H_2_O_bidest_ from that of the silver solution. The amount of Ag^+^ ions increased consecutively over time and was 1.8 ppm, 3.5 ppm, 4.1 ppm, 7.9 ppm, 9.2 ppm and 10.9 ppm at 8 min, 13 min, 18 min, 33 min, 43 min and 60 min, respectively, whereas no Ag^+^ ions were detectable before pulsing (Figure 1C). The total silver concentration consisting of Ag^+^ ions and unloaded silver particles was measured by ICP-OES. Compared to distilled water, 14 ppm total silver was detected 60 min after pulsing (Figure 1D). These data suggest that in addition to the measured value of 10.9 ppm Ag^+^ ions (compare Figure 1C), 3.1 ppm unloaded silver particles are present in the silver solution. For all subsequent experiments, the total silver concentration is given in ppm, as measured by ICP-OES. Finally, the size of the AgNPs was determined by TEM, which indicated an average particle core size of 9.04 nm, 60 min after pulsing (Figure 1E). This particle size was expected because it is within the published size of nanosilver, which ranges from 1 to 100 nm [17]. The cytotoxicity of our nanosilver was confirmed by the treatment of *E. coli* OP50 bacteria with different silver concentrations in the range from 1.4 ppm to 7 ppm. The OD_600_ was measured in 60-min intervals by the use of a spectrophotometer over a period of 8 h. The lowest AgNP concentration of 1.4 ppm markedly inhibited bacterial growth, which was enhanced at a concentration of 2.8 ppm and completely inhibited at a concentration of 4.2 ppm and higher (Figure 1F).

To address the cytotoxicity of AgNPs to human, benign, immortalized cell lines, we treated pancreatic ductal CRL-4023 cells and liver stellate LX-2 cells with AgNPs at final concentrations of 0.7 ppm, 1.4 ppm and 2.1 ppm. The controls were left untreated or were treated with H_2_O_bidest_ alone. Twenty-four hours later, viability was detected by MTT assay. Whereas CRL-4023 cells were totally resistant to either AgNP concentration, the viability of LX-2 cells was dose-dependently inhibited to 85%, 75% and 60% with 0.7 ppm, 1.4 ppm and 2.1 ppm AgNPs, respectively (Figure 1G). In contrast, the treatment of PDAC cell lines with AgNPs totally downregulated the viability of the BxPc-3, BxGEM, PANC-1 and MIA-PaCa2 cells, already at a AgNP concentration of 1.4 ppm and higher, whereas a lower concentration of 0.7 ppm was not effective in any cell line (Figure 1H). These results suggest that PDAC cells exhibit a much higher sensitivity to AgNPs than nonmalignant cells.

### 3.2. High AgNP Concentrations Are Toxic to Liver and Inhibit Embryonal Development

**Figure 2:**
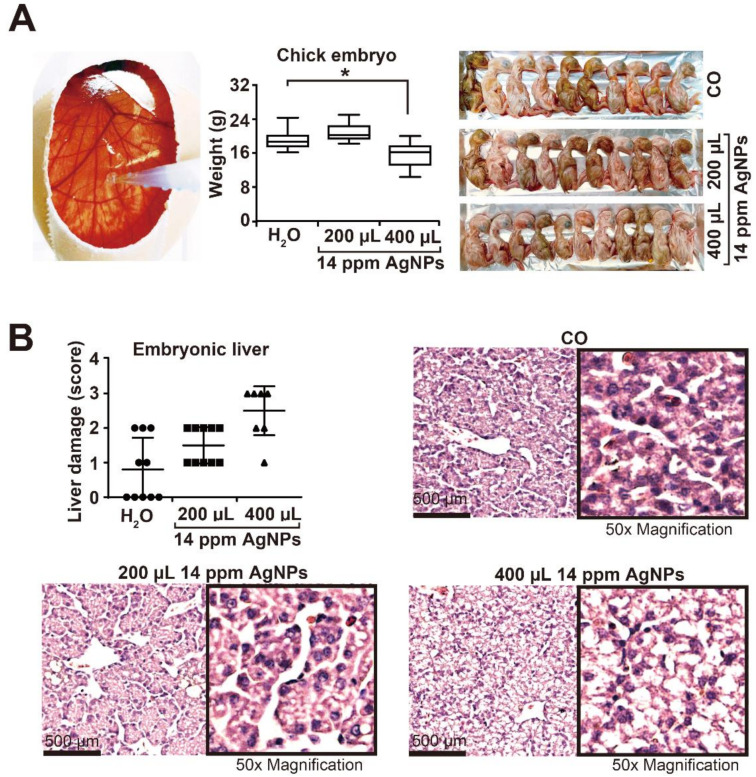
High AgNP concentrations are toxic to the liver and delay embryonal development. (**A**) Fertilized chicken eggs were prepared as described in the Materials and Methods. On day 12 of embryonal development, 400 µL H_2_O_bidest_ (CO) or 200 µL or 400 µL of a 14 ppm AgNP solution was dropped into the CAM twice daily until day 18. Then, the chick embryos were humanely euthanized, and the weight of the chicks in each group was evaluated. The mean weight per group (*n* = 10) ±SD and the photodocumented chicks are shown. (**B**) The embryonal livers were resected and stained with H&E, followed by microscopic evaluation. Liver damage was evaluated by the degree of cell morphology destruction of 10 vision fields per embryonic liver section (*n* = 10) by the following scoring system: 0: normal liver; 1: slightly damaged liver; 2: moderately damaged liver; and 3: severely damaged liver. The mean values ± SD and representative staining at 400× magnification, with 50× higher magnifications inside the boxes, are shown. * *p* < 0.05.

To obtain knowledge about the side effects of higher AgNP concentrations in vivo, we dropped nanosilver onto the CAM of fertilized chicken eggs. Starting at day 12 of chick development, 200 µL or 400 µL of a 14 ppm AgNP solution, or 400 µL H_2_O_bidest_ only in the control, were dropped twice daily to the CAM (Figure 2A). On day 18 of development, the eggs were opened, and the chicks were sacrificed and weighed. The mean chick weight of 20 g did not change upon treatment with 200 µL AgNPs or H_2_O alone, whereas 400 µL AgNPs significantly reduced the weight to approximately 16 g. In addition, the embryonal livers were resected, and deparaffinized tissue sections were stained with H&E, followed by immunohistochemistry and microscopic evaluation. We found that only the high amount of 400 µL AgNPs, but not the lower amount of 200 µL, led to a condensed liver structure that resembled necrotic tissue (Figure 2B). The degree of liver damage was microscopically evaluated in 10 vision fields per embryonic liver section by two blinded examiners with expertise in liver pathology. For quantitative evaluation, we used a scoring system, which is described in the figure legend.

### 3.3. α-Lipoic Acid Protects Nonmalignant Cells but Not PDAC Cells from AgNP-Induced Cytotoxicity

**Figure 3:**
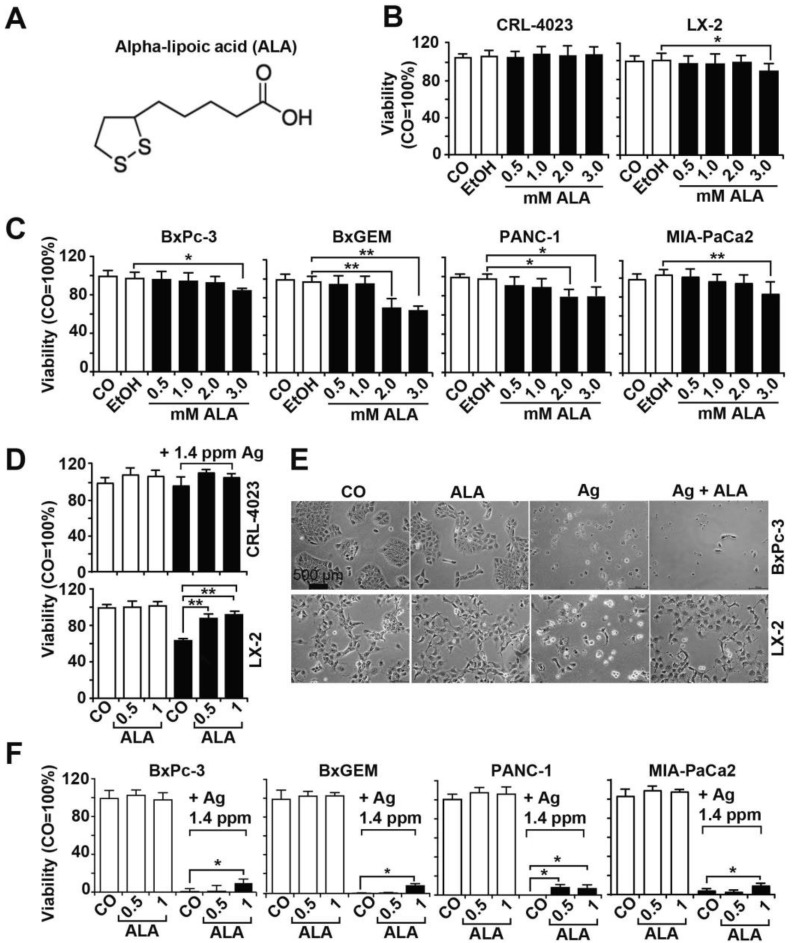
α-Lipoic acid protects nonmalignant cells from AgNP-induced toxicity but not PDAC cells. (**A**) Molecular structure of α-lipoic acid (ALA) with two sulfur atoms at the C6 and C8 positions, which are connected by a disulfide bond. (**B**) Nonmalignant CRL-4023 and LX-2 cells at a concentration of 5 × 10^3^ in 100 µL cell culture medium per well were seeded into 96-well plates and cultured for 24 h. Then, the cells were cultured by adding final concentrations of 0.5, 1, 2 and 3 mM α-lipoic acid to the cell culture medium. The controls were left untreated (CO), or 1.5 µL 99% EtOH was added. Twenty-four hours later, viability was evaluated by MTT assay. (**C**) Likewise, PDAC cell lines at a concentration of 5 × 10^3^ in a 100 µL cell culture medium per well were seeded in 96-well plates. Twenty-four hours later, the cells were treated and evaluated as described above. (**D**) CRL-4023 and LX-2 cells were incubated in 100 µL cell culture medium with final concentrations of 1.4 ppm AgNPs w and w/o 0.5 mM α-lipoic acid or 1 mM α-lipoic acid per well in 96-well plates. The viability was assessed 24 h later by MTT assay. (**E**) Representative images of the morphological changes observed microscopically in LX-2 and BxPc-3 cells at 400× magnification are shown. The scale bar represents 500 µm. (**F**) PDAC cells at a concentration of 5 × 10^3^ in 100 µL cell culture medium per well were seeded in 96-well plates. Twenty-four hours later, the cells were treated and evaluated as described above. The data for the MTT assay analysis were obtained from at least three independent experiments, and the controls were set to 100%. * *p* < 0.05, ** *p* < 0.01.

To avoid silver-induced side effects, we evaluated the suitability of the natural antioxidant and organosulfur component α-lipoic acid (Figure 3A), which is produced by plants, animals and humans, and is commercially available as a supplement [35]. Nonmalignant CRL-4023 and LX-2 cells were treated with α-lipoic acid at concentrations of 0.5 mM, 1 mM, 2 mM and 3 mM because these concentrations have recently been used for experimental treatment of breast cancer cells [36]. Twenty-four hours later, viability was determined by MTT assay. Both cell lines were largely resistant to any concentration; only the viability of LX-2 cells was slight but was significantly decreased by the highest concentration of 3 mM α-lipoic acid from 100% in the control to 90% (Figure 3B). Similar results were obtained by treatment of BxPc-3 and MIA-PaCa2 cells with α-lipoic acid, whereas BxGEM and PANC-1 were even more sensitive because their viability was already decreased at a α-lipoic acid concentration of 2 mM (Figure 3C). Since α-lipoic acid concentrations of 2 mM and higher seem to be slightly toxic, we used α-lipoic acid concentrations of 0.5 and 1 mM in subsequent experiments. Upon combined treatment of CRL-4023 and LX-2 cells with AgNPs and α-lipoic acid, the silver-induced toxicity was nearly completely abolished (Figure 3D,E). In contrast, the cotreatment of PDAC cells with AgNPs and α-lipoic acid only minimally interfered with the therapeutic silver toxicity, as the AgNPs nearly completely eliminated all cancer cells within 24 h, despite the presence of α-lipoic acid (Figure 3E).

### 3.4. α-Lipoic Acid Inhibits AgNP-Induced Mitochondrial Damage and ROS Formation

**Figure 4:**
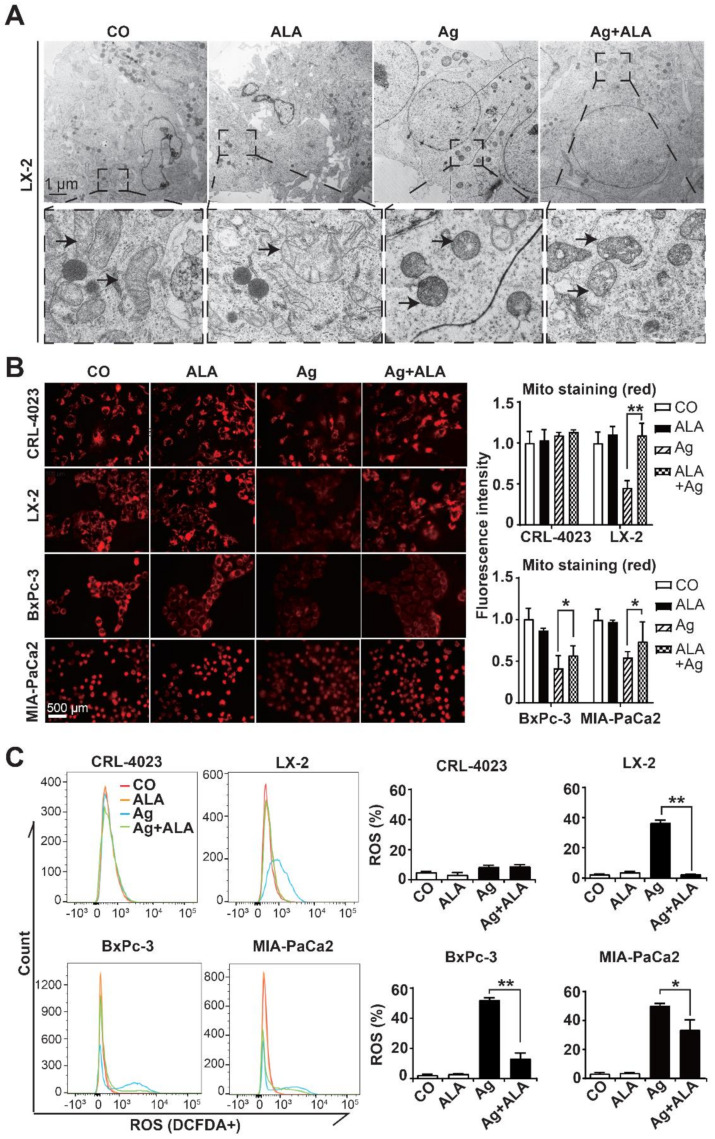
α-Lipoic acid rescues AgNP-induced mitochondrial damage and ROS formation in non-malignant but only partially rescues in PDAC cells. (**A**) LX-2 cells at a concentration of 5 × 10^3^ in 100 µL cell culture medium per well were seeded in 96-well plates. Twenty-four hours later, the cells were treated by adding 10 µL of a 14 ppm AgNP stock solution to the cell culture medium, resulting in a final working concentration of 1.4 ppm AgNPs. Likewise, 0.5 µL of a 200 mM α-lipoic acid (ALA) stock solution was added, resulting in a final concentration of 1 mM, as indicated. Control cells were left untreated (CO). Twenty-four hours later, the cells were prepared for TEM, followed by ultramicroscopic evaluation. Representative images are shown above, and 30× magnifications are shown below. The scale bar indicates 1 µm. The arrows mark the mitochondria. (**B**) CRL-4023, LX-2, BxPc-3 and MIA-PaCa2 at a concentration of 5 × 10^4^ in a 1 mL cell culture medium per well were seeded in 24-well plates. Twenty-four hours later, the cells were incubated in a 1 mL medium with 1.4 ppm AgNPs w and w/o 1 mM α-lipoic acid (ALA) at final concentrations or were left untreated in the control (CO). Twenty-four hours later, the cells were stained by adding 2 µL of the mitochondria-specific red fluorescent dye CytoPainter to the cell culture medium. After half an hour, 10 vision fields were randomly selected from each group and examined using a Leica DMRB fluorescence microscope at 400× magnification. Representative images are shown on the left, and the scale bar indicates 500 µm. The intensity of the red fluorescence was quantified by ImageJ software. Diagrams with the obtained mean data ± SD are shown on the right. (**C**) CRL-4023, LX-2, BxPc-3 and MIA-PaCa2 cells were seeded into 6-well plates at a density of 1.2 × 10^5^ per well. Twenty-four hours later, the cells were treated with final concentrations of 1.4 ppm AgNPs, 1 mM α-lipoic acid (ALA) or both together, or were left untreated (CO), followed by incubation for an additional 24 h. Then, the cells were incubated in medium with a final concentration of 10 µM H2DCFDA for 0.5 h. The fluorescence of DCF, which reflects the oxidized form of H2DCFDA, was measured with a FACSCanto II flow cytometer, and the excitation and emission wavelengths were 480 and 525 nm, respectively. Representative FACS histograms and diagrams with the obtained mean data ± SD are shown. * *p* < 0.05, ** *p* < 0.01.

To highlight the intracellular effects of AgNPs, we treated LX-2 cells with 1.4 ppm AgNPs, 1 mM α-lipoic acid or both agents together, or left the cells untreated in the control. Twenty-four hours later, the cell organelles were analyzed by TEM. The most obvious difference appeared in the mitochondria, which had a regular tubular shape in the untreated or α-lipoic acid-treated cells, whereas AgNPs induced a structural change to a globular, condensed shape (Figure 4A), which is consistent with the loss of the mitochondrial membrane potential [37]. In contrast, the combined treatment with AgNPs and α-lipoic acid largely prevented the loss of the membrane potential because the mitochondria still looked condensed but more tubular, which suggests that α-lipoic acid partly prevented AgNP-induced mitochondrial breakdown. To quantitatively measure the observed morphological changes, the cells were treated with the Mitochondrial Staining Reagent “Red-Cytopainter”, which is a fluorogenic probe to label the mitochondria of live cells and is retained in the mitochondria for a long time due to its cell-retaining group [38]. We detected the red fluorescence of this dye by fluorescence microscopy (Ex/Em = 350/490 nm). For quantification, we measured the fluorescence intensity using ImageJ in 10 vision fields per image of each group and calculated the mean fluorescence intensities and standard deviations. While the exposure of CRL-4023 cells to AgNPs, α-lipoic acid or both together did not change the fluorescence intensity (Figure 4B), the exposure of LX-2 cells to AgNPs significantly inhibited the fluorescence intensity, which indicates the loss of mitochondrial function. However, cotreatment with α-lipoic acid significantly prevented the inhibition of fluorescence intensity, most likely by preventing silver-induced toxicity. In contrast, α-lipoic acid only marginally restored the fluorescence intensity and thus silver toxicity in BxPc-3 and MIA-PaCa2 cells. These results reflect our former data obtained by MTT assays, where mitochondrial function is detected by the reduction of a tetrazolium component (MTT) into an insoluble formazan product by functional mitochondria [39]. We detected higher mitochondrial damage in PDAC cells because less formazan was measured (compare Figure 1G,H and Figure 3D–F).

To further highlight silver-induced mitochondrial damage and mitochondrial rescue by α-lipoic acid, we measured ROS formation. After treatment of nonmalignant cells and PDAC cell lines with AgNPs, α-lipoic acid or both together, we detected ROS by the use of the 2′,7′-dichlorofluorescein diacetate (H2DCFDA) assay and FACS analysis. Whereas α-lipoic acid alone did not increase DCFDA fluorescence, AgNPs induced a significant increase in DCFDA fluorescence and thus ROS formation in LX-2, BxPc-3 and MIA-PaCa2 cells but not in CRL-4023 cells (Figure 4C). Quantitatively, AgNPs induced 40% ROS formation in LX-2 cells and approximately 50% ROS formation in PDAC cells. Most importantly, the combination of AgNPs with α-lipoic acid completely abolished the elevated ROS formation in LX-2 cells but to a lower extent in PDAC cells, whereas pronounced ROS formation in CRL-4023 cells was not detectable. These data again suggest a higher sensitivity of malignant cells to AgNPs.

### 3.5. α-Lipoic Acid Reduces AgNP-Induced Cell Death

**Figure 5:**
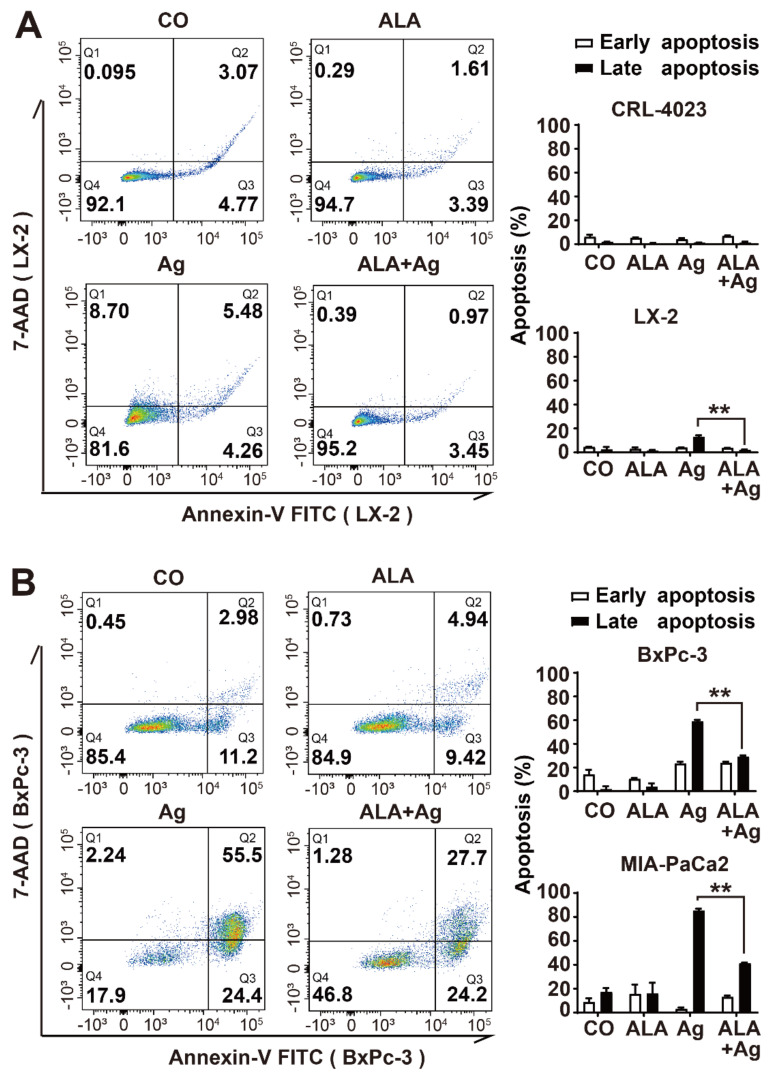
α-Lipoic acid inhibits AgNP-induced cell death in nonmalignant but not in PDAC cells. (**A**) CRL-4023 and LX-2 cells were seeded into 6-well plates at a density of 5 × 10^4^/mL per well. Twenty-four hours later, the cells were treated with 1.4 ppm AgNPs w and w/o 1 mM α-lipoic acid (ALA) at final concentrations or were left untreated in the control, followed by incubation for an additional 24 h. Then, the cells were harvested and incubated in 100 µL Annexin V Binding Buffer with 1 µM Annexin V-FITC for 0.5 h at room temperature. After washing with PBS, the cells were suspended and incubated in 300 µL Annexin V Binding Buffer supplemented with 2 µL 7-aminoactinomycin (7-AAD) for 20 min at room temperature. The procedure was protected from light. The fluorescence of Annexin V (excitation/emission: 494/518 nm) and 7-AAD (excitation/emission: 488/647 nm), which reflect apoptosis and necrosis, respectively, was measured with a FACSCanto II flow cytometer. Representative FACS plots of LX-2 are shown, and the diagrams of CRL-4023 and LX-2 with the obtained mean data ± SD are shown on the right. (**B**) The PDAC cell lines BxPc-3 and MIA-PaCa2 were treated and evaluated as described above. Representative FACS plots of BxPc-3 are shown, and the diagrams of BxPc-3 and MIA-PaCa2 with the obtained mean data ± SD are shown on the right. ** *p* < 0.01.

To evaluate the involvement of cell death in AgNP-induced toxicity, we treated CRL-4023, LX-2, BxPc-3 and MIAPaCa2 cells with 1.4 ppm AgNPs, 1 mM α-lipoic acid or both agents together, or left the cells untreated in the controls. Twenty-four hours later, the cells were stained with Annexin V-FITC for detection of phosphatidylserine exposure on the plasma membrane of cells undergoing early apoptosis and with the DNA-intercalating agent 7-actinomycin D (7-ADD) for detection of damaged DNA, which occurs in late apoptosis or necrosis. Then, the specific fluorescence signals were measured by FACS analysis. Whereas the fluorescence intensities in CRL-4023 did not increase noticeably with any treatment, the 7-AAD signal in LX-2 cells significantly increased to 15% upon silver treatment but was completely inhibited by α-lipoic acid cotreatment, as shown by representative FACS histogram blots and diagrams with the mean data and standard deviations (Figure 5A). AgNPs induced even higher rates of 7-AAD-positive cells, of 60% and 80%, in BxPc-3 and MIA-PaCa2 cells, respectively, which was significantly but not completely downregulated by α-lipoic acid cotreatment (Figure 5B). Because more 7-AAD-positive cells than annexin V-FITC-positive cells were found, we assume that AgNPs induce necrosis rather than apoptosis.

### 3.6. α-Lipoic Acid Precipitates Silver Particles and Ions

**Figure 6:**
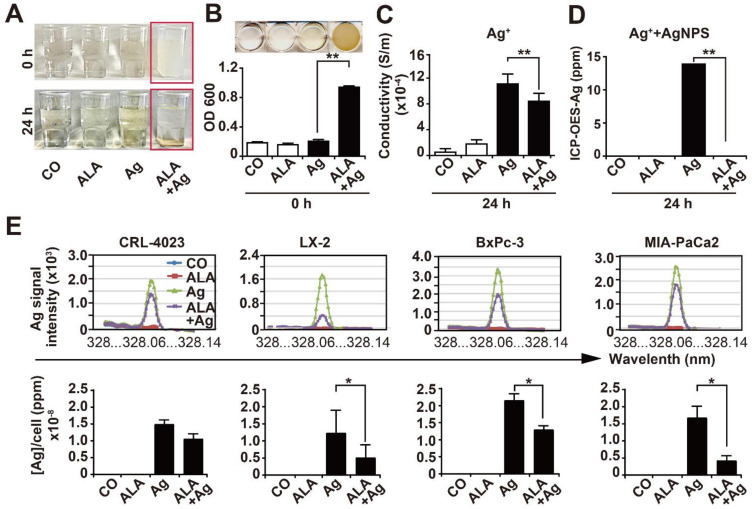
α-Lipoic acid chelates AgNPs and Ag ions. (**A**) A 20 mL H_2_O_bidest_ solution containing final concentrations of 10 mM α-lipoic acid and 14 ppm AgNPs was prepared (ALA + Ag). Likewise, 20 mL H_2_O_bidest_ alone, H_2_O_bidest_ containing a final concentration of 10 mM α-lipoic acid (ALA) or the final concentration of 14 ppm AgNPs (Ag) were prepared. Twenty-four hours later, representative images were taken. (**B**) The optical density of each aqueous solution was measured at 600 nm by a Biotek EL800 microplate reader, and the mean data ± SD are shown in a diagram. (**C**) Likewise, the presence of Ag^+^ ions was indirectly detected via conductivity measurements in each aqueous solution. The experiments were measured by the use of an HP8753C network analyzer in combination with an HP86046 A S-parameter test set and analyzed as described in the Materials and Methods. The data obtained from at least three independent experiments and results are shown as Siemens per meter (S/m) ± SDs. (**D**) The concentration of total silver (AgNPs and Ag^+^ ions) was measured by ICP-OES, and the mean concentrations are shown as ppm ± SD. (**E**) CRL-4023, LX-2, BxPc-3 and MIA-PaCa2 cells were seeded into cell culture dishes at a density of 8 × 10^4^ mL and incubated for 24 h. Then, the cells were precultured with a final concentration of 1 mM α-lipoic acid (ALA). After incubation for an additional 24 h, the medium of the ALA precultured cells was changed, and the cells were treated with a final concentration of 1.4 ppm AgNPs for 24 h or were left untreated in the control. The cells were dissolved in 65% HNO_3_ for 24 h, and the AgNP concentration was determined by ICP-OES. The data obtained from at least three independent experiments and the diagrams with the obtained mean data ± SD are shown. * *p* < 0.05, ** *p* < 0.01.

Because α-lipoic acid is described to form complexes with metal ions [31], we wanted to know whether the precipitation of AgNPs by α-lipoic acid may contribute to the observed protective effect. We mixed 10 mM α-lipoic acid with 14 ppm AgNPs to a total volume of 20 mL aqueous solution and observed that the AgNP aqueous solution immediately became cloudy after adding α-lipoic acid (Figure 6A). This could be confirmed by the measurement of the optical density at OD_600_, which significantly increased after adding α-lipoic acid to the AgNP solution (Figure 6B). Twenty-four hours later, the cloudy fog was sedimented at the bottom of the vial, which was associated with a loss of conductivity (Figure 6C) and a total loss of AgNPs, as detected by ICP-OES (Figure 6D). These data suggest neutralization of AgNPs by complex formation with α-lipoic acid. To further highlight the chelating activity of α-lipoic acid in living cells, we pretreated CRL-4023, LX-2, BxPc-3 and MIA-PaCa2 cells with 1 mM α-lipoic acid for 24 h, followed by incubation of the cells with 1.4 ppm AgNPs, as indicated, and ICP-OES analysis. AgNP treatment resulted in a significant increase in intracellular silver, which was strongly inhibited by cotreatment with α-lipoic acid, whereas α-lipoic acid alone or H_2_O_bidest_ in the control had no effect, as shown by representative spectroscopy histograms and diagrams with the means and standard deviations (Figure 6E). These results demonstrate that α-lipoic acid possesses excellent silver chelating properties.

### 3.7. α-Lipoic Acid Prevents AgNP-Induced Side Effects In Vivo but Does Not Affect the Therapeutic Efficacy of AgNPs on Tumor Growth

**Figure 7:**
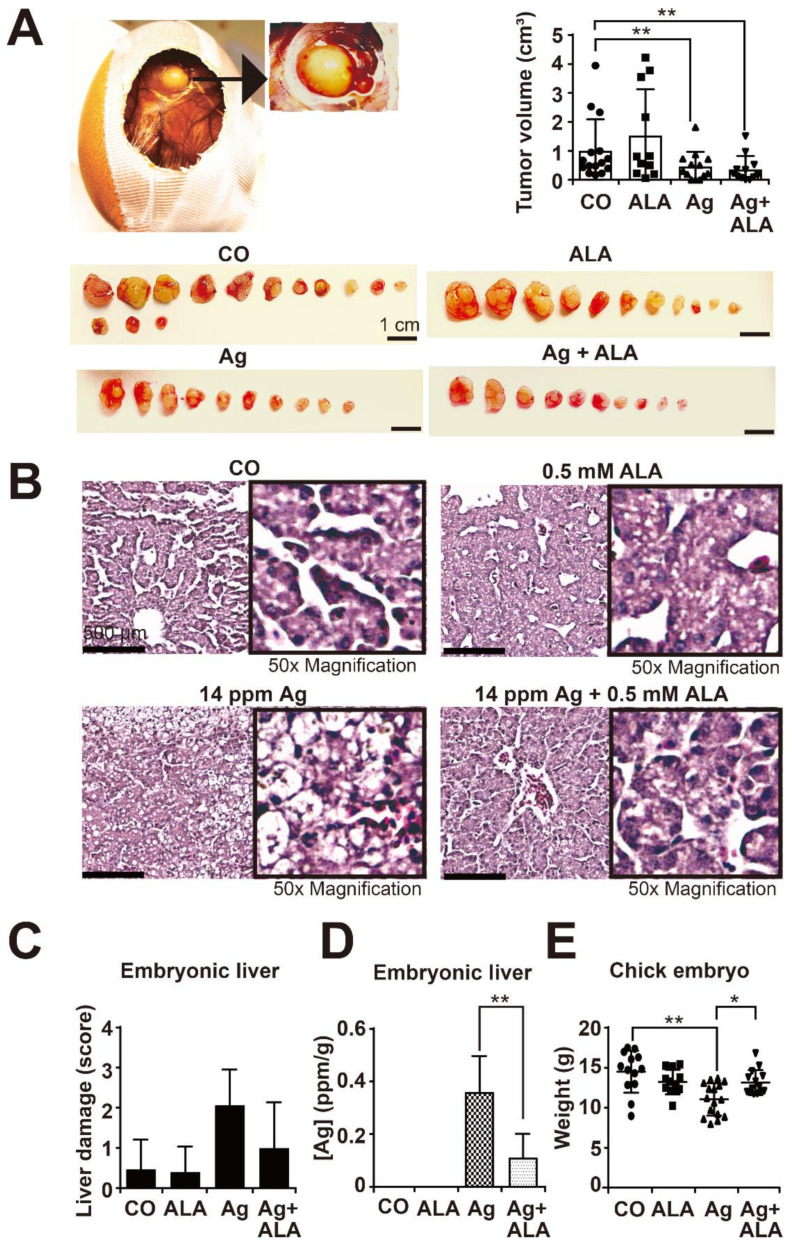
α-Lipoic acid prevents AgNP-induced liver toxicity and embryonal weight loss without affecting the inhibition of PDAC xenograft growth. (**A**) Fertilized chicken eggs were prepared for xenotransplantation as described in the Materials and Methods, and on day 9 of development, 10^6^ MIA-PaCa2 cells in 50 µL Matrigel^TM^ were transplanted onto the CAM. A representative image of a tumor xenograft at day 18 of chick development and a magnification thereof (black arrow) is shown on the upper left. On day 12 of embryonal development, 400 µL H_2_O_bidest_ (CO), 400 µL of a 14 ppm AgNP solution, 400 µL of a 0.5 mM α-lipoic acid (ALA) solution or 400 µL of a complex of 14 ppm AgNPs with 0.5 mM ALA was separately dropped onto the CAM vessels that supplied the xenograft tumors g until day 18. Tumor xenografts were resected on day 18 and photographed, and images of the tumor sizes of each group are shown below. The scale bar indicates 1 cm. The individual tumor volumes in cm^3^ and the mean volumes of each group ± SD are presented on the upper right. Please note that the images show the two-dimensional size, but the diagram on the left the three-dimensional tumor volume, and both do not necessarily match. (**B**) The embryonal livers were resected, stained with H&E and microscopically evaluated. Representative staining liver tissue of each group is shown at 400× magnification, and 50× higher magnifications are shown on the right. (**C**) The level of liver damage was quantified by the degree of cell morphology destruction in 10 randomly chosen vision fields of each embryonic liver section (*n* = 10) by the following scoring system: high, medium, low and absent damage were scored as 3, 2, 1 and 0, respectively. (**D**) The amount of silver accumulation in each gram of liver was measured by ICP-OES. (**E**) The evaluation of the weight of the chicks in each group with the obtained mean data ± SD is shown. CO (*n* = 13), ALA (*n* = 11), Ag (*n* = 16) and Ag + ALA (*n* = 15). * *p* < 0.05, ** *p* < 0.01.

To evaluate the in vivo functionality, we used the transplantation of tumor cells to the CAM of fertilized chicken eggs. This avian xenograft model is a naturally immunodeficient system, the tumor microenvironment is similar to that of immunodeficient mice, and it is well suited for short-term evaluation of tumor growth for up to 10 days [22]. MIA-PaCa2 cells were transplanted onto the CAM on day 9 of embryonic development, which is a time point of chick development at which the embryonic blood vessel system is dense enough to support xenograft growth. From developmental day 12 to day 17, 400 µL of a 14 ppm AgNP solution, 400 µL of a 0.5 mM α-lipoic acid solution or both together were dropped twice daily onto the CAM vessels that supplied the Mia-PaCa2 xenograft tumors. Control eggs received 400 µL H_2_O_bidest_ twice a day. The chick embryos were humanely euthanized at day 18 of development followed by resection of tumor xenografts and embryonic livers. A representative image of a tumor xenograft growing in the egg is shown, along with images of each resected tumor xenograft and a diagram with the individual tumor volumes, the means and standard deviations (Figure 7A). The results show that AgNPs alone significantly reduced the mean tumor size, while α-lipoic acid alone had no effect and did not affect the AgNP-mediated inhibition of tumor xenograft growth in the combination treatment.

To examine the side effects, tissue sections of the embryonic livers were stained with H&E, followed by immunohistochemistry. Upon AgNP treatment, the morphology of the embryonic livers exhibited signs of ballooning degeneration, which was described as enlarged hepatocytes with a wispy cleared cytoplasm and a condensed structure resembling necrotic tissue (Figure 7B). However, the combination of AgNPs with α-lipoic acid prevented this morphology of a damaged liver. The results were quantified by the use of the scoring system described in the figure legend, and the mean values with standard deviations are shown in a diagram (Figure 7C). Whereas AgNPs increased liver toxicity, the combination with α-lipoic acid reduced liver toxicity, although these differences were not statistically significant. In addition, the tissue from 10 livers of each treatment group was minced, followed by the measurement of the silver concentration by ICP-OES. The detected amounts of total silver were significantly enhanced by AgNP treatment compared to the controls but significantly downregulated by α-lipoic acid cotreatment (Figure 7D). Accordingly, AgNPs significantly reduced the mean weight of chick embryos, and this side effect was significantly reversed by α-lipoic acid cotreatment (Figure 7E). These results indicate that α-lipoic acid cotreatment strongly reduces AgNP-mediated silver accumulation and side effects but does not affect the therapeutic efficacy of AgNPs on tumor growth.

### 3.8. AgNPs Inhibits BCAT1 Expression, Which Is Rescued by α-Lipoic Acid

**Figure 8:**
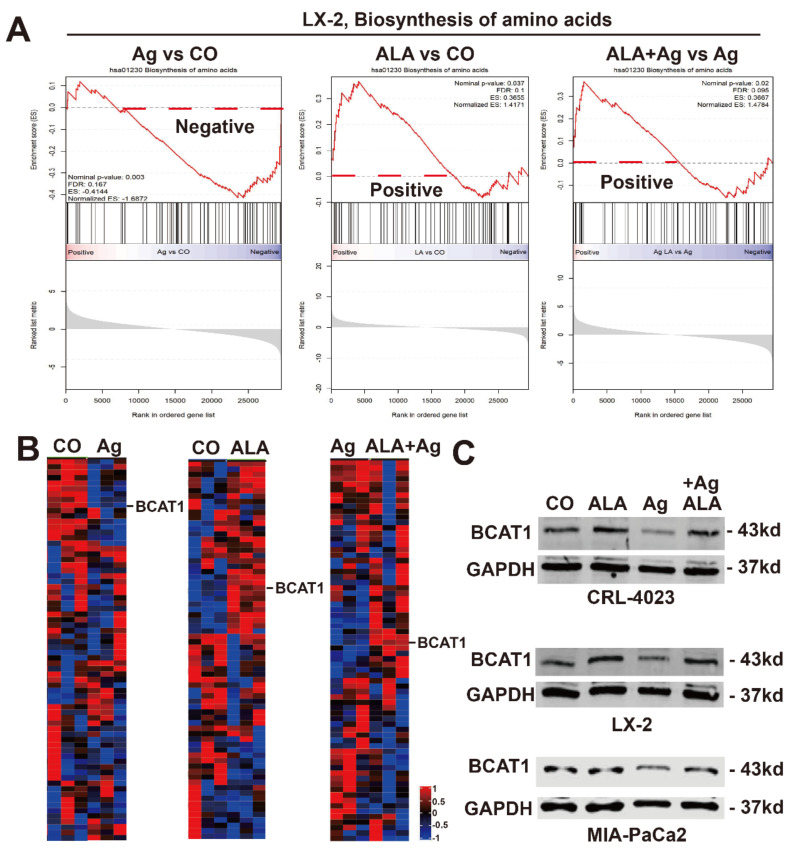
α-Lipoic acid rescues AgNP-induced downregulation of BCAT1 in nonmalignant but not in PDAC cells. (**A**) LX-2 cells were seeded into 6-well plates at a density of 5 × 10^4^/mL per well. Twenty-four hours later, the cells were treated with 1.4 ppm AgNPs w and w/o 1 mM α-lipoic acid (ALA) at final concentrations or were left untreated in the control, followed by incubation for an additional 24 h. Then, the cells were harvested, and mRNA was isolated by using the RNeasy Mini Kit, followed by microarray analysis with Clariom™ D Assays in triplicate and bioinformatic evaluation. The results of GSEA demonstrated that biosynthesis of amino acids was downregulated by AgNPs but upregulated by α-lipoic acid cotreatment or α-lipoic acid alone, which is presented as enrichment plots. (**B**) The results of the heat map reveal 71 genes related to the biosynthesis of amino acids, which were significantly up- or downregulated genes (red: high expression; blue: low expression). The scale from 1 to −1 indicates the relative expression. The expression of the branched chain amino acid transaminase 1 (BCAT1) is indicated. (**C**) CRL-4023, LX-2, BxPc-3 and MIA-PaCa2 cells were seeded into 6-well plates at a density of 5 × 10^4^/mL per well and cultured for 24 h. Then, the cells were treated with 1.4 ppm AgNPs w and w/o 1 mM α-lipoic acid at final concentrations or were left untreated in the control, followed by incubation for an additional 24 h. The proteins were harvested, and the expression of BCAT1 was detected by Western blot analysis. GAPDH served as a control to ensure equal loading conditions. The protein sizes in kilodaltons (kDa) are given on the right.

To further elucidate the interplay between α-lipoic acid and AgNPs, we treated LX-2 cells with AgNPs and α-lipoic acid alone, or both together, or left the cells untreated in the control. Then, the RNA was harvested and examined by an mRNA profiling array, which was analyzed by GSEA, a pathway enrichment method that evaluates microarray data at the level of gene sets. Based on the bioinformatics evaluation, we observed that the biosynthesis of amino acids was downregulated by AgNPs but upregulated by α-lipoic acid cotreatment or α-lipoic acid alone, presented as enrichment plots (Figure 8A). In addition, uisng KEGG analysis, whose results are presented as a heat map, we identified 71 genes related to the biosynthesis of amino acids (Figure 8B). Of particular interest was the branched chain amino acid transaminase 1 (BCAT1) gene, which was downregulated by AgNPs, while the α-lipoic acid cotreatment or α-lipoic acid alone upregulated the expression of BCAT1. BCAT1 is involved in transamination-dependent glutamate synthesis, which is required to produce glutathione, which is capable of preventing damage caused by ROS [40]. The AgNP-induced downregulation of BCAT1 and the rescue of its expression by cotreatment with α-lipoic acid were confirmed by Western blot analysis and treatment of CRL-4023, LX-2 and MIA-PaCa2 cells (Figure 8C).

## 4. Discussion

Here, we demonstrate that the natural antioxidant α-lipoic acid is able to reverse AgNP-induced side effects on benign cells and tissues while preserving the therapeutic effect on PDAC cells. Our data suggest the cotreatment of tumor cells with nanosilver in the presence of α-lipoic acid as a safe new treatment option for pancreatic cancer patients.

We produced AgNPs with an IDEAL-Pulser Basic S silver generator, which resulted in approximately 2/3 silver ions and 1/3 unloaded silver particles with an average size of 9.04 nm. By the use of 200 mL H_2_O_bidest_, at least 99.99% pure silver electrodes, and a pulsing time of 60 min, we obtained a silver concentration of 14 ppm. This concentration is different from that given by the manufacturer, who found a 100 ppm AgNP concentration after pulsing H_2_O_dest_ for 60. This difference may be due to the aggregation of AgNPs [41] during the production process, resulting in a large loss of AgNPs after filtration with a 0.2 µm syringe filter, as we did, or it may be caused by the different methods of measurement.

By treating *E. coli* OP50 bacteria with different AgNP concentrations, we ensured antibacterial activity and found that bacterial growth was totally inhibited by a 7 ppm AgNP solution, which is consistent with the results of Yoojin Choi et al. [42]. This antimicrobial effect was most likely mediated by AgNP-induced damage to the bacterial cell membrane because AgNPs generate pits and gaps in the bacterial cell membrane and thereby destroy its permeability [43].

The AgNP-mediated eradication of *E. coli* implies that the intake of colloidal silver may damage the gut microbiome. Indeed, a recent study reported that dietary AgNPs, which because of their antimicrobial properties are added by the food industry to consumer products or are used for coating components from food processing machines or plastic food packaging, led to an estimated intake of dietary silver at 70–90 μg/day, as estimated for 2009, and such an AgNP concentration disturbed the gut microbiota in mice [44]. This is interesting because an altered gut microbiome contributes to tumor biology, malignant transformation, tumor progression and the therapy response of cancer [45]. However, this may be a double-edged sword because AgNPs on the one hand may eradicate an unfavorably altered, tumor-contributing gut microbiome, and on the other hand, they may destroy a healthy gut microbiome and thereby contribute to tumorigenesis. To take speculation to the extreme, one could even assume that AgNPs may be able to damage the tumor microbiome, which has been detected and characterized in several tumor entities [46], although its role in tumorigenesis is currently unclear.

By treating benign cells and PDAC cell lines with AgNPs, we detected that a working concentration of 1.4 ppm AgNPs almost completely inhibited the viability of malignant cells, whereas benign cells largely resisted the inhibition of viability. This finding of selective toxicity of AgNPs toward malignant cells corresponds to recent data that demonstrated lysis of the cell membrane of the human lung cancer cell line A549 by 3 ppm AgNPs, whereas no obvious leakage of the cell membrane of nonmalignant L132 lung cells occurred [47]. We demonstrated that high AgNP concentrations of 400 µL of a 14 ppm AgNP solution twice a day for a period of 6 days reduced the weight of chick embryos, accumulated in the liver and was liver toxic. Our data are underlined by previous studies, which showed that AgNPs increase fetal mortality and developmental inhibition after pregnant rats were orally administered 10 mg AgNPs per kilogram body weight [17]. Furthermore, Lee et al. found that AgNPs induce piecemeal necrosis and chronic inflammation in the liver, along with silver accumulation/deposition, in rats after intraperitoneal injection of AgNPs at a concentration of 500 mg/kg [48]. If we convert our AgNP in vivo concentration of 400 µL of a 14 ppm (=14 mg/L) AgNP solution twice daily to body weight, 800 µL AgNPs correspond to 4% of the body weight of a chick embryo of 20 g. Related to a 70 kg person, this amount is comparable to the daily intake of 2.8 L of a 14 ppm (=14 mg/L) AgNP solution, which corresponds to a total silver amount of 39 mg AgNPs/70 kg ingested daily.

Mechanistically, we observed that AgNPs impaired mitochondrial function by altering the shape and number of mitochondria and by increasing the ROS levels, which converged to necrotic cell death. AgNPs may enter the mitochondria by endocytosis and distribution to the cytoplasm and nucleus through intracellular trafficking, as suggested [49]. Silver ions can be continually released from the surface of AgNP particles and adhere to the cell wall and cytoplasmic membrane because of electrostatic attraction and affinity to sulfur proteins [50]. Adherent ions enhance the permeability of the cytoplasmic membrane, and the uptake of free silver ions deactivates respiratory enzymes and generates ROS, whose overproduction provokes disruption of the cell membrane and DNA modification [51]. Most importantly, we observed that AgNPs induced more mitochondrial dysfunction and ROS formation, followed by more necrotic cell death, in PDAC cells than in nonmalignant cells. In general, we observed that PDAC cells respond more sensitively than benign cells to AgNPs.

The major challenge in the clinical use of AgNPs for tumor therapy is to effectively deliver silver nanoparticles and ions to the target tumor tissue without inducing adverse effects and silver deposition in nonmalignant tissues. Our results illustrated that the coadministration of α-lipoic acid significantly decreased AgNP-induced ROS formation and protected the mitochondrial membrane potential of nonmalignant cells. Our results are consistent with those of recent publications, which demonstrated that α-lipoic acid and its metabolite, dihydrolipoic acid (DHLA), are capable of scavenging a variety of ROS, including hydroxyl radicals, hypochlorous acid and singlet oxygen [52,53,54]. This is because α-lipoic acid indirectly plays a role in maintaining the cellular antioxidant status by inducing the synthesis of endogenous low molecular weight antioxidants or antioxidant enzymes, such as increasing the intracellular ascorbate levels [27] and augmenting the cellular glutathione pool [55].

By gene array analysis and bioinformatics, we found that AgNPs inhibited the expression of branched chain amino acid transaminase 1 (BCAT1), thereby reducing transamination-dependent glutamate synthesis [56]. The synthesis of glutamate makes an important contribution to maintaining the relatively large intracellular glutathione pool, which is capable of preventing damage caused by ROS [40]. Then, we observed that cotreatment with α-lipoic acid completely rescued BCAT1 downregulation by increasing the expression of BCAT1 in nonmalignant cells, indicating that α-lipoic acid reverses AgNP-induced glutamate depletion and thereby protects against ROS formation. However, compared with the control group, the expression of BCAT1 in the MIA-PaCa2 cell line was still significantly downregulated after cotreatment with α-lipoic acid, and there was no expression of BCAT1 in BxPc-3 cells. This may be due to cancer-specific BCAT1 expression, as suggested by Mayers et al., who demonstrated that PDAC patient tissues expressed lower BCAT1 levels than normal tissues [56]. Therefore, the low basal BCAT1 expression in PDAC cells may be one major reason for the observed failure of α-lipoic acid to protect malignant cells from AgNP-induced toxicity. In addition, oncogenic KRAS mutations are commonly seen in most human pancreatic tumors [57] and are associated with increased glucose and glutamate consumption to support anabolic processes, including nucleotide, lipid and nonessential amino acid biosynthesis [58]. These data explain why α-lipoic acid mainly rescued nonmalignant cells from AgNP-induced cytotoxicity but not PDAC cells. To our knowledge, our finding is the first report describing the crosstalk/interactions between α-lipoic acid and BCAT1.

In addition to being an antioxidant, α-lipoic acid is described as a chelator of redox-active metal ions with a preference for Pb^2+^, Zn^2+^ and Cu^2+^ [31]. Here, we show that AgNPs and Ag^+^ ions are strongly chelated by α-lipoic acid, and the complexes were excreted from the cells. Then, we proceeded to test the efficacy of α-lipoic acid and found no side effects on nonmalignant cells at concentrations up to 3 mM. This is in line with previous results of a clinical trial, where patients were treated with high lipoic acid concentrations of 800 mg/day for 4 years, and no side effects were observed [59]. In contrast, α-lipoic acid even improved health outcomes in overall healthy individuals and in patients affected by other diseases [60]. Additionally, our findings of the safety of α-lipoic acid in nonmalignant cells and tissues and its putative suitability for pancreatic cancer treatment are underlined by a recent study of the treatment of rats xenografted with pancreatic tumors and treated with nab-paclitaxel [61]. α-Lipoic acid was applied at concentrations of 15, 30 and 60 mg/kg and prevented oxidative stress and peripheral neuropathy without diminishing the body weight or the chemotherapeutic effect on tumor growth. Our in vivo results demonstrate that 0.5 mM α-lipoic acid prevented side effects without diminishing the AgNP effect on xenograft growth. Calculated to a human being, considering a relative blood volume of 4000 mL, this suggests the intake of 412 mg α-lipoic acid/day. This concentration is realistic and corresponds to the α-lipoic acid concentration of 800 mg/day, which was applied to patients for 4 years without any negative side effects [59]. Interestingly, α-lipoic acid has been clinically used in Germany as a pharmaceutical drug for the treatment of diabetic polyneuropathy for more than 50 years [62].

Finally, we verified our concept in vivo by the use of tumor xenotransplantation to fertilized chicken eggs. This avian model is an excellent alternative to experiments performed on mammals, such as mice [63], and has been evaluated in several of our recent studies [64,65]. One major advantage of the chicken egg model is its natural immunodeficiency because immunity occurs only after hatching [66]. Thus, cells from other species or tissues are accepted to be similar to immune-compromised mice. In this model, xenografts are transplanted into the chorioallantoic membrane (CAM) between days 8–9 of development because at that time point, the network of blood vessels is dense enough to support the growth of a tumor xenograft. An additional advantage of CAM xenotransplantation is fast tumor growth, which starts between 2 and 5 days after transplantation. Using this model, we confirmed our in vitro data, and because the AgNPs alone inhibited xenograft growth of the PDAC xenografts, cotreatment with α-lipoic acid prevented liver cytotoxicity and developmental disturbance without interfering with the AgNP-induced inhibition of tumor growth.

## 5. Conclusions

In conclusion, we have demonstrated an easy, simple and patient-friendly approach to ingest therapeutic AgNPs in tumor diseases and avoid the toxic side effects to normal tissues. However, our study has limitations because we mainly performed in vitro studies. Although we verified the results by in vivo xenotransplantation, our data cannot be transferred one-to-one to the patient without confirmation in clinical studies. Nevertheless, our data are promising and point to a more successful therapy for pancreatic cancer and other tumor entities in the future.

## Data Availability

The datasets supporting the conclusions of this article and its supplemental files are included within the article and thus are available.

## Abbreviations

7-ADD	7-aminoactinomycin
CAM	Chorioallantoic membrane
H2DCFDA	2′,7′-dichlorodihydrofluorescein diacetate
ICP-OES	Inductively coupled plasma-optical emission spectrometry
FDR	False discovery rate
GSEA	Gene Set Enrichment Analysis
AgNPs	Silver nanoparticles
PDAC	Pancreatic ductal adenocarcinoma
(MTT)	3-(4,5-dimethylthiazol-2-yl)-2,5-diphenyltetrazolium bromide
TEM	Transmission electron microscopy

## References

[1] Siegel R.L., Miller K.D., Jemal A. (2020). Cancer statistics, 2020. CA Cancer J. Clin..

[2] Gurunathan S., Qasim M., Park C., Yoo H., Kim J.-H., Hong K. (2018). Cytotoxic Potential and Molecular Pathway Analysis of Silver Nanoparticles in Human Colon Cancer Cells HCT116. Int. J. Mol. Sci..

[3] Chugh H., Sood D., Chandra I., Tomar V., Dhawan G., Chandra R. (2018). Role of gold and silver nanoparticles in cancer nano-medicine. Artif. Cells Nanomed. Biotechnol..

[4] Barcińska E., Wierzbicka J., Zauszkiewicz-Pawlak A., Jacewicz D., Dabrowska A., Inkielewicz-Stepniak I. (2018). Role of Oxidative and Nitro-Oxidative Damage in Silver Nanoparticles Cytotoxic Effect against Human Pancreatic Ductal Adenocarcinoma Cells. Oxidative Med. Cell. Longev..

[5] Mohler J.S., Sim W., Blaskovich M.A.T., Cooper M.A., Ziora Z.M. (2018). Silver bullets: A new lustre on an old antimicrobial agent. Biotechnol. Adv..

[6] Merkl P., Long S., McInerney G.M., Sotiriou G.A. (2021). Antiviral Activity of Silver, Copper Oxide and Zinc Oxide Nanoparticle Coatings against SARS-CoV-2. Nanomaterials.

[7] Amato E., Diaz-Fernandez Y.A., Taglietti A., Pallavicini P., Pasotti L., Cucca L., Milanese C., Grisoli P., Dacarro C., Fernandez-Hechavarria J.M. (2011). Synthesis, characterization and antibacterial activity against Gram positive and Gram negative bacteria of biomimetically coated silver nanoparticles. Langmuir.

[8] Griffith R.D., Simmons B.J., Bray F.N., Falto-Aizpurua L.A., Yazdani Abyaneh M.A., Nouri K. (2015). 1064 nm Q-switched Nd:YAG laser for the treatment of Argyria: A systematic review. J. Eur. Acad. Dermatol. Venereol..

[9] Nowack B., Krug H.F., Height M. (2011). 120 years of nanosilver history: Implications for policy makers. Environ. Sci. Technol..

[10] Loh J.V., Percival S.L., Woods E.J., Williams N.J., Cochrane C.A. (2009). Silver resistance in MRSA isolated from wound and nasal sources in humans and animals. Int. Wound J..

[11] Anuj S.A., Gajera H.P., Hirpara D.G., Golakiya B.A. (2019). Interruption in membrane permeability of drug-resistant *Staphylococcus aureus* with cationic particles of nanosilver. Eur. J. Pharm. Sci..

[12] Ip M., Lui S.L., Poon V.K., Lung I., Burd A. (2006). Antimicrobial activities of silver dressings: An in vitro comparison. J. Med. Microbiol..

[13] Yang F., Ren Z., Chai Q., Cui G., Jiang L., Chen H., Feng Z., Chen X., Ji J., Zhou L. (2016). A novel biliary stent coated with silver nanoparticles prolongs the unobstructed period and survival via anti-bacterial activity. Sci. Rep..

[14] Park W., Kim K.Y., Kang J.M., Ryu D.S., Kim D.H., Song H.Y., Kim S.H., Lee S.O., Park J.H. (2020). Metallic Stent Mesh Coated with Silver Nanoparticles Suppresses Stent-Induced Tissue Hyperplasia and Biliary Sludge in the Rabbit Extrahepatic Bile Duct. Pharmaceutics.

[15] Rogers K.R., Navratilova J., Stefaniak A., Bowers L., Knepp A.K., Al-Abed S.R., Potter P., Gitipour A., Radwan I., Nelson C. (2018). Characterization of engineered nanoparticles in commercially available spray disinfectant products advertised to contain colloidal silver. Sci. Total Environ..

[16] Hadrup N., Lam H.R. (2014). Oral toxicity of silver ions, silver nanoparticles and colloidal silver—A review. Regul. Toxicol. Pharmacol..

[17] Ema M., Okuda H., Gamo M., Honda K. (2017). A review of reproductive and developmental toxicity of silver nanoparticles in laboratory animals. Reprod. Toxicol..

[18] Li Y., Qin T., Ingle T., Yan J., He W., Yin J.-J., Chen T. (2017). Differential genotoxicity mechanisms of silver nanoparticles and silver ions. Arch. Toxicol..

[19] Hamida R.S., Albasher G., Bin-Meferij M.M. (2020). Oxidative Stress and Apoptotic Responses Elicited by Nostoc-Synthesized Silver Nanoparticles against Different Cancer Cell Lines. Cancers.

[20] Satapathy S.R., Mohapatra P., Preet R., Das D., Sarkar B., Choudhuri T., Wyatt M.D., Kundu C.N. (2013). Silver-based nanoparticles induce apoptosis in human colon cancer cells mediated through p53. Nanomedicine.

[21] Kwon H.B., Lee J.H., Lee S.H., Lee A.Y., Choi J.S., Ahn Y.S. (2009). A case of argyria following colloidal silver ingestion. Ann. Dermatol..

[22] Bracey N.A., Zipursky J.S., Juurlink D.N. (2018). Argyria caused by chronic ingestion of silver. Can. Med. Assoc. J..

[23] White J.M., Powell A.M., Brady K., Russell-Jones R. (2003). Severe generalized argyria secondary to ingestion of colloidal silver protein. Clin. Exp. Dermatol..

[24] Shelley W.B., Shelley E.D., Burmeister V. (1987). Argyria: The intradermal “photograph,” a manifestation of passive photosensitivity. J. Am. Acad. Dermatol..

[25] Loeschner K., Hadrup N., Qvortrup K., Larsen A., Gao X., Vogel U., Mortensen A., Lam H.R., Larsen E.H. (2011). Distribution of silver in rats following 28 days of repeated oral exposure to silver nanoparticles or silver acetate. Part. Fibre Toxicol..

[26] Mathur P., Jha S., Ramteke S., Jain N.K. (2018). Pharmaceutical aspects of silver nanoparticles. Artif. Cells Nanomed. Biotechnol..

[27] Shay K.P., Moreau R.F., Smith E.J., Smith A.R., Hagen T.M. (2009). Alpha-lipoic acid as a dietary supplement: Molecular mechanisms and therapeutic potential. Biochim. Biophys. Acta.

[28] Patrick L. (2002). Mercury toxicity and antioxidants: Part 1: Role of glutathione and alpha-lipoic acid in the treatment of mercury toxicity. Altern. Med. Rev..

[29] Smirnova J., Kabin E., Järving I., Bragina O., Tõugu V., Plitz T., Palumaa P. (2018). Copper(I)-binding properties of de-coppering drugs for the treatment of Wilson disease. α-Lipoic acid as a potential anti-copper agent. Sci. Rep..

[30] Yoon F.H., Gardner S.L., Danjoux C., Morton G., Cheung P., Choo R. (2008). Testosterone Recovery after Prolonged Androgen Suppression in Patients with Prostate Cancer. J. Urol..

[31] Ou P., Tritschler H.J., Wolff S.P. (1995). Thioctic (lipoic) acid: A therapeutic metal-chelating antioxidant?. Biochem. Pharmacol..

[32] Cotton G.C., Gee C., Jude A., Duncan W.J., Abdelmoneim D., Coates D.E. (2019). Efficacy and safety of alpha lipoic acid-capped silver nanoparticles for oral applications. R. Soc. Chem..

[33] Fan P., Liu L., Yin Y., Zhao Z., Zhang Y., Amponsah P.S., Xiao X., Bauer N., Abukiwan A., Nwaeburu C.C. (2016). MicroRNA-101-3p reverses gemcitabine resistance by inhibition of ribonucleotide reductase M1 in pancreatic cancer. Cancer Lett..

[34] Schaefer M., Gross W., Ackemann J., Gebhard M.M. (2002). The complex dielectric spectrum of heart tissue during ischemia. Bioelectrochemistry.

[35] Salehi B., Berkay Yılmaz Y., Antika G., Boyunegmez Tumer T., Fawzi Mahomoodally M., Lobine D., Akram M., Riaz M., Capanoglu E., Sharopov F. (2019). Insights on the use of alpha-lipoic acid for therapeutic purposes. Biomolecules.

[36] Tripathy J., Tripathy A., Thangaraju M., Suar M., Elangovan S. (2018). α-Lipoic acid inhibits the migration and invasion of breast cancer cells through inhibition of TGFβ signaling. Life Sci..

[37] Miyazono Y., Hirashima S., Ishihara N., Kusukawa J., Nakamura K.I., Ohta K. (2018). Uncoupled mitochondria quickly shorten along their long axis to form indented spheroids, instead of rings, in a fission-independent manner. Sci. Rep..

[38] Kong J.-N., Zhu Z., Itokazu Y., Wang G., Dinkins M.B., Zhong L., Lin H.-P., Elsherbini A., Leanhart S., Jiang X. (2018). Novel function of ceramide for regulation of mitochondrial ATP release in astrocytes. J. Lipid Res..

[39] Rai Y., Pathak R., Kumari N., Sah D.K., Pandey S., Kalra N., Soni R., Dwarakanath B.S., Bhatt A.N. (2018). Mitochondrial biogenesis and metabolic hyperactivation limits the application of MTT assay in the estimation of radiation induced growth inhibition. Sci. Rep..

[40] McBrayer S.K., Mayers J.R., DiNatale G.J., Shi D.D., Khanal J., Chakraborty A.A., Sarosiek K.A., Briggs K.J., Robbins A.K., Sewastianik T. (2018). Transaminase Inhibition by 2-Hydroxyglutarate Impairs Glutamate Biosynthesis and Redox Homeostasis in Glioma. Cell.

[41] Wiley B., Sun Y., Mayers B., Xia Y. (2005). Shape-controlled synthesis of metal nanostructures: The case of silver. Chemistry.

[42] Choi Y., Kim H.-A., Kim K.-W., Lee B.-T. (2018). Comparative toxicity of silver nanoparticles and silver ions to *Escherichia coli*. J. Environ. Sci..

[43] Li W.-R., Xie X.-B., Shi Q.-S., Zeng H.-Y., Ou-Yang Y.-S., Chen Y.-B. (2010). Antibacterial activity and mechanism of silver nanoparticles on *Escherichia coli*. Appl. Microbiol. Biotechnol..

[44] van den Brule S., Ambroise J., Lecloux H., Levard C., Soulas R., De Temmerman P.J., Palmai-Pallag M., Marbaix E., Lison D. (2016). Dietary silver nanoparticles can disturb the gut microbiota in mice. Part. Fibre Toxicol..

[45] Helmink B.A., Khan M.A.W., Hermann A., Gopalakrishnan V., Wargo J.A. (2019). The microbiome, cancer, and cancer therapy. Nat. Med..

[46] Nejman D., Livyatan I., Fuks G., Gavert N., Zwang Y., Geller L.T., Rotter-Maskowitz A., Weiser R., Mallel G., Gigi E. (2020). The human tumor microbiome is composed of tumor type-specific intracellular bacteria. Science.

[47] Gurunathan S., Jeong J.-K., Han J.W., Zhang X.-F., Park J.H., Kim J.-H. (2015). Multidimensional effects of biologically synthesized silver nanoparticles in Helicobacter pylori, Helicobacter felis, and human lung (L132) and lung carcinoma A549 cells. Nanoscale Res. Lett..

[48] Lee T.-Y., Liu M.-S., Huang L.-J., Lue S.-I., Lin L.-C., Kwan A.-L., Yang R.-C. (2013). Bioenergetic failure correlates with autophagy and apoptosis in rat liver following silver nanoparticle intraperitoneal administration. Part. Fibre Toxicol..

[49] Greulich C., Diendorf J., Simon T., Eggeler G., Epple M., Köller M. (2011). Uptake and intracellular distribution of silver nanoparticles in human mesenchymal stem cells. Acta Biomater..

[50] Bapat R.A., Chaubal T.V., Joshi C.P., Bapat P.R., Choudhury H., Pandey M., Gorain B., Kesharwani P. (2018). An overview of application of silver nanoparticles for biomaterials in dentistry. Mater. Sci. Eng. C Mater. Biol. Appl..

[51] Yin I.X., Zhang J., Zhao I.S., Mei M.L., Li Q., Chu C.H. (2020). The Antibacterial Mechanism of Silver Nanoparticles and Its Application in Dentistry. Int. J. Nanomed..

[52] Scott B.C., Aruoma O.I., Evans P.J., O’Neill C., van der Vliet A., Cross C.E., Tritschler H., Halliwell B. (1994). Lipoic and dihydrolipoic acids as antioxidants. A critical evaluation. Free Radic. Res..

[53] Bjørklund G., Aaseth J., Crisponi G., Rahman M.M., Chirumbolo S. (2019). Insights on alpha lipoic and dihydrolipoic acids as promising scavengers of oxidative stress and possible chelators in mercury toxicology. J. Inorg. Biochem..

[54] Devasagayam T.P., Di Mascio P., Kaiser S., Sies H. (1991). Singlet oxygen induced single-strand breaks in plasmid pBR322 DNA: The enhancing effect of thiols. Biochim. Biophys. Acta.

[55] Camiolo G., Tibullo D., Giallongo C., Romano A., Parrinello N.L., Musumeci G., Di Rosa M., Vicario N., Brundo M.V., Amenta F. (2019). α-Lipoic Acid Reduces Iron-induced Toxicity and Oxidative Stress in a Model of Iron Overload. Int. J. Mol. Sci..

[56] Mayers J.R., Torrence M.E., Danai L.V., Papagiannakopoulos T., Davidson S.M., Bauer M.R., Lau A.N., Ji B.W., Dixit P.D., Hosios A.M. (2016). Tissue of origin dictates branched-chain amino acid metabolism in mutant Kras-driven cancers. Science.

[57] Kleeff J., Korc M., Apte M., La Vecchia C., Johnson C.D., Biankin A.V., Neale R.E., Tempero M., Tuveson D.A., Hruban R.H. (2016). Pancreatic cancer. Nat. Rev. Dis. Primers.

[58] White E. (2013). Exploiting the bad eating habits of Ras-driven cancers. Genes Dev..

[59] Derosa G., D’Angelo A., Preti P., Maffioli P. (2020). Safety and Efficacy of Alpha Lipoic Acid During 4 Years of Observation: A Retrospective, Clinical Trial in Healthy Subjects in Primary Prevention. Drug Des. Dev. Ther..

[60] Fogacci F., Rizzo M., Krogager C., Kennedy C., Georges C.M.G., Knežević T., Liberopoulos E., Vallée A., Pérez-Martínez P., Wenstedt E.F.E. (2020). Safety Evaluation of α-Lipoic Acid Supplementation: A Systematic Review and Meta-Analysis of Randomized Placebo-Controlled Clinical Studies. Antioxidants.

[61] Sun H., Guo X., Wang Z., Wang P., Zhang Z., Dong J., Zhuang R., Zhou Y., Ma G., Cai W. (2019). Alphalipoic Acid Prevents Oxidative Stress and Peripheral Neuropathy in Nab-Paclitaxel-Treated Rats through the Nrf2 Signalling Pathway. Oxidative Med. Cell. Longev..

[62] Ziegler D., Reljanovic M., Mehnert H., Gries F.A. (1999). Alpha-lipoic acid in the treatment of diabetic polyneuropathy in Germany: Current evidence from clinical trials. Exp. Clin. Endocrinol. Diabetes.

[63] Aleksandrowicz E., Herr I. (2015). Ethical euthanasia and short-term anesthesia of the chick embryo. ALTEX.

[64] Yin L., Xiao X., Georgikou C., Yin Y., Liu L., Karakhanova S., Luo Y., Gladkich J., Fellenberg J., Sticht C. (2019). MicroRNA-365a-3p inhibits c-Rel-mediated NF-κB signaling and the progression of pancreatic cancer. Cancer Lett..

[65] Luo Y., Yan B., Liu L., Yin L., Ji H., An X., Gladkich J., Qi Z., de La Torre C., Herr I. (2021). Sulforaphane Inhibits the Expression of Long Noncoding RNA H19 and Its Target APOBEC3G and Thereby Pancreatic Cancer Progression. Cancers.

[66] Ribatti D. (2017). The chick embryo chorioallantoic membrane (CAM) assay. Reprod. Toxicol..

